# New Bicyclic Sesquiterpene and Labdane Diterpenes from the Culture Extract of the Sea Grass-Derived Fungus *Penicillium verruculosum* KUFA1509 †

**DOI:** 10.3390/md24060205

**Published:** 2026-06-10

**Authors:** Diana I. C. Pinho, Tida Dethoup, Ruchiluk Rattarom, Emília Sousa, Salar Hafez-Ghoran, Artur M. S. Silva, Luís Gales, Anake Kijjoa

**Affiliations:** 1 School of Medicine and Biomedical Sciences Abel Salazar (ICBAS) and CIIMAR, Universidade do Porto, Rua de Jorge Viterbo Ferreira 228, 4050-313 Porto, Portugal; up201706356@edu.ff.up.pt; 2 Laboratório de Química Orgânica e Farmacêutica, Departamento de Ciências Químicas, Faculdade de Farmácia, Universidade do Porto and CIIMAR, Rua de Jorge Viterbo Ferreira 228, 4050-313 Porto, Portugal; esousa@ff.up.pt; 3 Department of Plant Pathology, Faculty of Agriculture, Kasetsart University, Bangkok 10240, Maha Sarakham, Thailand; agrtdd@ku.ac.th; 4 Pharmaceutical Chemistry and Natural Product Research Unit, Faculty of Pharmacy, Mahasarakham University, Kantharawichai 44150, Maha Sarakham, Thailand; rujiluk.r@msu.ac.th; 5 Laboratory for Functional Foods and Human Health, Center for Excellence in Post-Harvest Technologies, North Carolina Agricultural and Technical State University, North Carolina Research Campus, 500 Laureate Way, Kannapolis, NC 28081, USA; s_hafezghoran@yahoo.com; 6 Departamento de Química & QOPNA, Universidade de Aveiro, 3810-193 Aveiro, Portugal; artur.silva@ua.pt; 7 School of Medicine and Biomedical Sciences Abel Salazar (ICBAS) and Instituto de Biologia Molecular e Celular (i3S-IBMC), Universidade do Porto, Rua de Jorge Viterbo Ferreira 228, 4050-313 Porto, Portugal

**Keywords:** *Penicillium verruculosum* KUFA1509, marine-derived fungus, sea grass, verruculosic acid, labdane diterpenes, *nor*-seco-labdane diterpene, nitric oxide inhibitory activity, RAW264.7 macrophages

## Abstract

An unreported bicyclic sesquiterpene acid, verruculosic acid (**1**), was isolated together with the previously reported labdane diterpenes, (+)-agathic acid (**2a**) and hypoxyterpenoid A (**2b**), one 3-*nor*-2,3-seco-labdane, penioxalicin (**3**), and 5-carboxyphthalide (**4**), from a sea grass-associated fungus, *Penicillium verruculosum* KUFA1509. The structures of the isolated compounds were elucidated by detailed analyses of 1D and 2D NMR and HRMS data. The absolute configurations of the stereogenic carbons in **1** and **2a** were established by X-ray crystallography. The crystal structure of **2a**, which was obtained for the first time, was used to prove its structure and confirm its stereochemistry. The crystal structure of **3** was also obtained; however, the value of its flack parameter does not allow us to determine the absolute configuration. Compound **2b** exhibited stronger inhibitory activity than the positive control, diclofenac sodium, against LPS-induced nitric oxide (NO) production in RAW264.7 macrophages, while **1** and **2a** were slightly less active than the positive control. In contrast, **3** exhibited much weaker activity than **2a**. Compounds **1**–**4** were also assayed for antibacterial activity against reference and multidrug-resistant strains, but none exhibited antibacterial activity against the tested strains. Thus, the labdane skeleton could be considered as a potential scaffold for the development of anti-inflammatory agents through NO inhibition.

## 1. Introduction

The fungi of the genus *Penicillium* constitute an important part of marine microorganisms, contributing around 22% of secondary metabolites produced by marine-derived fungi [1]. Although polyketides are the most commonly isolated compounds, alkaloids, terpenoids, steroids and peptides are also the main classes of secondary metabolites produced by marine-derived *Penicillium* species [1,2,3]. Moreover, a myriad of unreported compounds, with relevant biological and pharmacological activities, continue to be isolated from marine-derived *Penicillium* fungi, thus making them very attractive for a research program in drug discovery [4]. For these reasons, numerous reviews on natural products isolated from marine-derived fungi of the genus *Penicillium* have been published [1,2,3,4,5].

Terpenoids are a relevant class of bioactive secondary metabolites produced by marine-derived *Penicillium* species [6,7]. Marine-derived *Penicillium* species produce diverse terpenoid classes, particularly sesquiterpenoids, diterpenoids, and meroterpenoids [8]. Sesquiterpenes represent the largest and most prolific group of marine fungal terpenoids, many of which exhibited interesting biological and pharmacological activities [8]. Ma et al. [9] described the isolation of three drimane sesquiterpenes, purpurides E–G, from a marine-derived *P. minioluteum* ZZ1657, which was isolated from a sample of marine sediment, collected from the East China Sea. Purpuride G exhibited antiproliferative activities against human glioma U251 and U87MG cells, with IC_50_ values of 4.49 and 10.9 µM, respectively. Gou et al. [10] isolated two drimane sesquiterpenes, *viz.* (4*S*,5*R*,9*S*,10*R*)-11,13-dihydroxydrim-7-en-6-one and (4*S*,5*R*,9*S*,10*R*)-11-hydroxy-13-carboxydrim-7-en-6-one, from *Penicillium* sp. TW58-16, obtained from a hydrothermal vent sediment, collected from Kueishantao, Taiwan. (4*S*,5*R*,9*S*,10*R*)-11,13-Dihydroxydrim-7-en-6-one exhibited strong *α*-glucosidase inhibitory effects, with inhibition rates of 35.4% (the inhibition rates of the positive control, acarbose, was 34.9%). Zhang et al. [11] reported the isolation of eremophilane-type sesquiterpenes, copteremophilanes A–J, from a marine sponge-associated *P. copticola*, isolated from the marine sponge, *Xestospongia testudinaria*, collected from Weizhou Island, China. Copteremophilane G showed a neuroprotective effect by increasing the viability of Aβ_25–35_-induced PC12 cells, whereas copteremophilane H exhibited a selective inhibition against human non-small cell lung cancer cells (A549). Another rare but interesting group of sesquiterpenes, isolated from marine-derived *Penicillum* species, are acorane-type sesquiterpenes. Structurally, they are bicyclic sesquiterpenes characterized by a unique spiro [4.5]decane skeleton. Zhang et al. [12] reported the isolation of 18 acorane-type sesquiterpenes, named bilaiaeacorenols A–R, from *P. bilaiae* F-28, which was collected from a deep-sea sediment (GPS 27.90 W, 6.43 S, depth of 5610 m) in the South Atlantic Ocean. Bilaiaeacorenol R exhibited a significant reduction in nitric oxide (NO) production in LPS-induced BV-2 macrophages in a dose-dependent manner. This compound also eliminated LPS-induced NF-κB in the nucleus of BV-2 microglial cells. Marine-derived fungi of the genus *Penicillium* are also an important source of diterpenes with novel structures and relevant biological activity such as cytotoxicity, antibacterial and enzyme inhibitory activities [7]. Although *Penicillium* fungi produced a large number of indole diterpenes [13], many diterpenes of complicated structures such as cyclopiane diterpenes have also been reported from marine-derived *Penicillium* species, especially from the deep-sea [14,15].

Some simple diterpenes such as primarane and labdane diterpenes have also been reported from *Penicillium* species. An example of these is a pimarane diterpene, named diaporthein C, isolated from a sea slug gut-derived *P. sclerotiorum* GZU-XW03-2. This compound showed an inhibitory effect against α-glucosidase, with an IC_50_ value of 282 µM, which is more potent than the positive control, acarbose (IC_50_ = 1330 µM) [16]. Despite the fact that the labdane-type diterpenoids are mainly produced by plants, and rarely found in fungi, Cheng et al. [17] described the isolation of 3β-hydroxyagathic acid, 3β-acetoxyagathic acid and agathic acid from *P. thomii* YPGA3, obtained from the deep-sea water, at a depth of 4500 m, in the Yap Trench (West Pacific Ocean). The same research group has also isolated a 19-*nor* labdane-type diterpenoid, named penitholabene, from the same fungal material. Penitholabene exhibited an inhibitory effect against α-glucosidase, with an IC_50_ value of 282 μM, which is more active than the positive control, acarbose (IC_50_ = 1.33 mM) [18].

The study of secondary metabolites from *P. verruculosum* (recently reclassified as *Talaromyces verruculosus*) is rather scarce [19]. A literature search of *P. verruculosum* revealed the chemical investigation of the color metabolites of *P. verruculosum* SG., obtained from samples taken from Kala pani forest in Khyber Pakhtunkhwa, Pakistan. Liquid chromatography-mass spectrometry (LCMS) and the fragmentation patterns revealed the presence of various polyketides such as monascin, monascorubrine, scirpentriol, verucine A, demethyl calcimycin, citrinadine, orevactaene and calcimycin. Moreover, phenazine-1-carboxylic acid, obtained as a crystal, was identified by the X-ray crystallographic analysis [20]. Despite the lack of investigation on its secondary metabolites, *P. verruculosum* is well-known for its value in biotechnology. For example, *P. verruculosum* B1-537 produces large amounts of secreted protein (up to 70 g/L), and is used for large-scale enzyme production. The high secretory capacity and the previously developed expression system, based on the strong inducible *cbh1* promoter of *P. verruculosum*, allow this fungus to be used as a platform for the expression of heterologous genes and large-scale production of enzyme preparations for technical and food purposes [21]. In addition to the major cellobiohydrolase I (CBHI), the *P. verruculosum* native secretome contains endogenous glucoamylase, pvGlaA, expressed under starch-induced conditions, suggesting that *P. verruculosum* B1-537 may be a potential glucoamylase producer for industrial applications [22].

A literature search of *Talaromyces verruculosus* revealed that Miao et al. [23] have reported the isolation of 3-(4-hydroxypentyl)-8-hydroxy-3,4-dihydroisocoumarin and (*E*)-3-(2,5-dioxo-3-(propan-2-ylidene)pyrrolidin-1-yl) acrylic acid from *P. verruculosum* YL-52, isolated from the rhizosphere soil of *Stellera chamaejasme* L., collected in the Qinling Mountains of Taibai town in Shaanxi Province in China. Very interestingly, a marine-derived *T. verruculosus*, isolated from the soft coral *Goniopora* sp., collected from Sanya, Hainan island, South China Sea, China, furnished the oligophenalenone dimer, verruculosin A, a new analog verruculosin B, together with bacillisporin F, duclauxin, and xenoclauxin [24].

Due to the lack of investigation of the secondary metabolites from *P. verruculosum*, especially from the marine environment, we have investigated the secondary metabolites produced by a marine-derived *P. verruculosum* KUFA1509, isolated from a sea grass, *Enhalus acoroides* (Family Hydrocharitaceae), which was collected from the coral reef at Hong Island of Krabi province, Southern Thailand.

## 2. Results and Discussion

**Figure 1:**
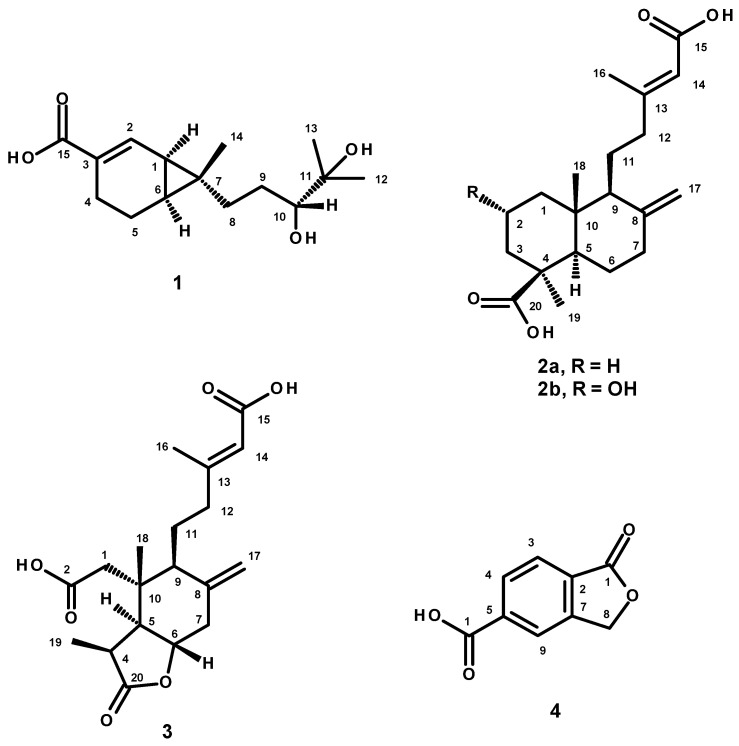
Structure of **1**–**4**.

The EtOAc crude extract of *P. verruculosum* KUFA1509 was first partitioned to CHCl_3_ and, after evaporation of the solvent by reduced pressure, the crude CHCl_3_ extract was obtained. Fractionation of the crude CHCl_3_ extract by column chromatography of silica gel, followed by purification by crystallization, preparative TLC and Sephadex LH-20 column, furnished an unreported bicyclic sesquiterpene acid, verruculosic acid (**1**), two labdane-type diterpenes, (+)-agathic acid (**2a**) and hypoxyterpenoid A (**2b**), a *nor*-seco-labdane-type diterpene, penioxalicin (**3**), and 5-carboxyphthalide (**4**) (Figure 1).

Compound **1** was initially isolated as a colorless viscous mass. However, after several attempts to crystallize in mixtures of CHCl_3_ and MeOH, white colorless crystals were formed. The ^1^H NMR spectrum (in DMSO-*d6*) of **1** (Table 1, Figure S1) exhibited a broad doublet of one proton at δ_H_ 7.11 (*J* = 5.3 Hz), a doublet of one proton at δ_H_ 3.03 (*J* = 8.9 Hz), several overlapping multiplets spanning from δ_H_ 1.5 to δ_H_ 2.3, two multiplets centered at δ_H_ 1.26 and 1.18, and three methyl singlets at δ_H_ 1.05, 1.00 and 0.82, respectively. The ^13^C NMR spectrum (Table 1, Figure S2) exhibited 15 carbon signals which, in combination with DEPT and HSQC spectra (Figure S4), can be categorized as one conjugated carboxylic acid or ester carbonyl (δ_C_ 168.5), one protonated sp^2^ carbon (δ_C_ 138.9), one non-protonated sp^2^ carbon (δ_C_ 127.7), one oxymethine sp^3^ (δ_C_ 77.7), one oxyquarternary sp^3^ (δ_C_ 72.1), one quarternary sp^3^ (δ_C_ 33.1), two methine sp^3^ (δ_C_ 25.0 and 22.9), four methylene sp^3^ (δ_C_ 40.5, 28.4, 21.9 and 17.2) and three methyl (δ_C_ 26.9, 24.9 and 13.5) carbons (Table 1, Figures S2 and S4). The existence of the 3,4-disubstituted cyclohex-1-ene-1-carboxylic acid was substantiated by HMBC correlations from H-2 (δ_H_ 7.11, d, *J* = 5.3 Hz) to CO-15 (δ_C_ 168.5), C-1 (δ_C_ 22.9) and C-6 (δ_C_ 25.0), H-4 (δ_H_ 1.78 m/2.28 m) to C-2 (δ_C_ 127.7) and C-3 (δ_C_ 138.9), and H-1 (δ_H_ 1.26 m) to C-3 (Table 1, Figure S5). Additionally, the COSY spectrum exhibited correlations from H-2 to H-1 and one of H-4 (δ_H_ 1.78 m) (Table 1, Figure S3). That the cyclohexene ring fused with a methylcyclopropane ring to form a 7-methylbicyclo [4.1.0]hept-2-ene system was substantiated by HMBC correlations from the methyl singlet at δ_H_ 0.82 (Me-14, δ_C_ 13.5) to C-1, C-6, C-7 (δ_C_ 33.1), H-1 and H-5 to C-7 (Table 1, Figure S5). Another substituent on C-7 is the 3,4-dihydroxy-4-methylpentyl group, which is evidenced by COSY correlations (Table 1, Figure S3) from H-10 (δ_H_ 3.03, d, *J* = 8.9 Hz/δ_C_ 77.7) to H-9 (δ_H_ 1.21 m and 1.66 m/δ_C_ 28.4) as well as HMBC correlations from H-10 to C-8 (δ_C_ 40.5), Me-14 to C-8, Me-12 (δ_H_ 1.05 m/δ_C_ 26.9) and Me-13 (δ_H_ 1.00 m/δ_C_ 24.9) to C-10 (δ_C_ 77.7), and C-11 (δ_C_ 72.1) (Table 1, Figure S5). This structure provides the molecular formula C_15_H_24_O_4_ (MW = 268). Intriguingly, the (+)-HRESIMS of **1** gave the *m/z* 269.1919 [M+H]^+^, calcd for C_15_H_24_O_4,_ 269.1753. Since the accurate mass (*m/z* 269.1919) showed the measured mass 61.7 ppm away from the calculated mass (*m/z* 269.1753), this error is far too large and hence the measured mass does not match the molecular formula C_15_H_24_O_4_. However, the (+)-HRESIMS showed a prominent peak at *m/z* 251.1654 [M-H_2_O+H]^+^, calcd for C_15_H_23_O_3,_ 251.1647 (Figure S28). This result indicated that the structure of **1** must contain a tertiary hydroxyl group, which is supported by the presence of the oxyquarternary sp^3^ carbon at δ_C_ 72.1. Moreover, the (+)-HRESIMS showed the base peak at *m/z* 233.1550, calcd for C_15_H_21_O_2_, 233.1542, which corresponds to the loss of a second H_2_O. Therefore, the planar structure of **1** is established as 7-(3,4-dihydroxy-4-methylpentyl)-7-methylbicyclo [4.1.0]hept-2-ene-3-carboxylic acid.

A literature search revealed that the planar structure of **1** is the same as that of the isomeric sesquiterpenes, sesquicaranoic acids A and B, isolated from the stems of *Illicium jiadifengpi*, collected in the Guangxi Province, China [25]. The absolute configurations at C-1, C-6, C-7 and C-10 of sesquicaranoic acid A were assigned by circular dichroism (CD) and Mo2(OAc)4-induced circular dichroism (ICD) as 1*S*, 6*R*, 7*S*, 10*S*. The assignment of 1*S*, 6*R*, 7*S* configurations was based on a negative Cotton effect at 217 nm (Δε-5.46) of the CD spectrum from the π → π* excitation of the α, β-unsaturated carboxylic acid chromophore, indicating the *S* configuration at C-1 on the basis of the allylic quasi-axial hydrogen rule, and further assigned the absolute configurations at C-6 and C-7 as 6*R* and 7*S*. Based on the empirical rule proposed by Snatzke, the positive Cotton effect at 310 nm (Δε + 7.23) in the ICD spectrum allowed the assignment of the configuration at C-10 as 10*S*. Surprisingly, the authors mistakenly named sesquicaranoic acid A as (1*S*,6*R*,7*S*)-7-((3*S*)-3,4-hydroxy-4-methyl)-7-methylbicyclo [4.1.0]hept-2-en-3-carboxylic acid. Since the ICD spectrum of sesquicaranoic acid B showed a negative Cotton effect at 311 nm (Δε-10.78), this compound was identified as a C-10 epimer of sesquicaranoic acid A, and the structure of sesquicaranoic acid B was also incorrectly named as (1*S*,6*R*,7*S*)-7-((3*R*)-3,4-hydroxy-4-methyl)-7-methylbicyclo [4.1.0]hept-2-en-3-carboxylic acid. It is also interesting to point out that sesquicaranoic acid A is levorotatory, with [α]^20^_D_-6.3 (MeOH), while sesquicaranoic acid B is dextrorotatory, with [α]^20^_D_ +45.9 (MeOH). Sesquicaranoic acid B was also isolated from the fungus *Penicillium* sp. LPFH-hzw-zw1, obtained from a sediment sample collected from Hangzhou Bay, China [26], and from a marine-derived fungal strain *P. janthinellum* JK07-5, collected from the Bohai Sea, China [27]. Additionally, sesquicaranoic acid C, an analog of sesquicaranoic A, was isolated from the pericarps of *Illicium difengpi*, collected from Jingxi County, Guangxi Province, China, by Ning et al. The differences between sesquicaranoic acid A and sesquicaranoic acid C are that the vicinal hydroxyl groups on C-10 and C-11 were replaced by a double bond between C-10 and C-11, and Me-13 in sesquicaranoic acid A was replaced by a methylformate group in sesquicaranoic acid C. The absolute configuration at C-1 in sesquicaranoic acid C was determined as 1*S* based on the negative Cotton effect at 213 nm in the CD spectrum, which led to further assignment of the absolute configurations at C-6 and C-7 as 6*R*, 7*S* [28].

**Figure 2:**
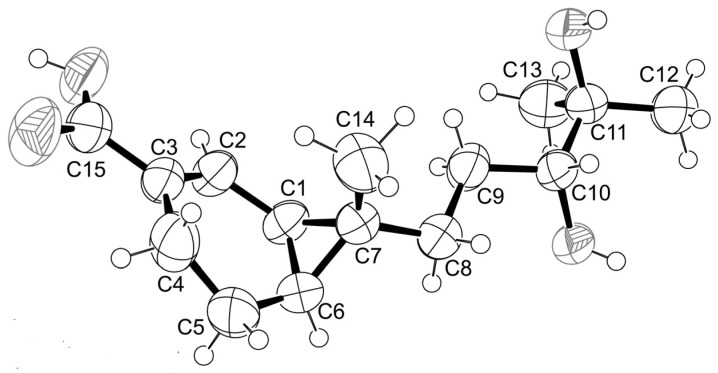
Ortep view of **1**.

Since **1** could be obtained as a suitable crystal for X-ray crystallographic analysis using X-ray diffractometer equipped with the CuKα radiation, the configurations at C-1, C-6, C-7 and C-10 were established unequivocally as 1*S*, 6*S*, 7*R*, 10*R.* The Ortep view of **1** is shown in Figure 2. Compound **1** was levorotatory, with [α]^20^_D_-160 (MeOH). Therefore, a complete structure of **1** is established as (1*S*,6*S*,7*R*)-7-((3*R*)-3,4-dihydroxymethylpentyl)-7-methylbicyclo [4.1.0]hept-2-ene-3-carboxylic acid. Compound **1** is thus a diastereomer of sesquicaranoic acids A and B. Since **1** has never been previously reported, it was named verruculosic acid.

Compound **2a** was isolated as white crystals. Analysis of its 1D and 2D NMR data (Table S1, Figures S7–S11) revealed that its planar structure is the same as that of agathic acid. Intriguingly, both enantiomers of agathic acid, i.e., (+)-agathic acid (a labdane diterpene) and (-)-agathic acid (an *ent*-labdane diterpene) occur in nature. B. R. Thomas described the isolation of (+)-agathic acid from the acetone-soluble bled resin of *Agathis australis* or the New Zealand kauri (kauri gum) in 1966. The compound was dextrorotatory, with [α]_D_ + 54º (CHCl_3_) [29]. (+)-Agathic acid was also isolated from aerial parts of *Chloranthus serratus,* collected in Guangdong Province, China. However, the authors reported only the ^13^C NMR data (in CD_3_OD) of (+)-agathic acid ([α]^20^_D_ + 48), and only the relative configurations of the stereogenic carbons were established by ROESY correlations but the absolute configurations were not determined [30]. Interestingly, (+)-agathic acid was also isolated from fungi. Zhou et al. described the isolation of (+)-agathic acid from a solid culture of the insect-pathogenic fungus, *Paecilomyces* sp. ACCC 37762, which was obtained from an unidentified Lepidopteran, collected in Hebei, China. The authors also reported the ^1^H and ^13^C NMR data of (+)-agathic acid (in CD_3_OD) and determined the relative configurations by ROESY correlations [31]. Furthermore, (+)-agathic acid was obtained by the in vitro biotransformation of isocupressic acid by incubation, under anaerobic conditions, with a ruminal fluid mixture. In addition, (+)-agathic acid was obtained by chemical conversion of isocupressic acid, and its identity was proved by ^1^H and ^13^C NMR spectral analysis as well as by optical rotation ([α]^20^_D_ +55.5, EtOH) [32]. Intriguingly, (+)-agathic acid was later reported from the marine-derived fungus, *Penicillium thomii* YPGA3, obtained from the deep-sea water at a depth of 4500 m in the Yap Trench (West Pacific Ocean) [17]. On the other hand, *ent*-agathic acid or (-)-agathic acid was reported from several sources. Zdero et al. reported the isolation of (-)-agathic acid from aerial parts of *Aristeguetia buddleaefolia* [33]. Moreover, (-)-agathic acid was isolated from the oleoresin of *Copaifera langsdorffii* [34] and *C. reticulata* [35]. Xin et al. described a synthesis of (-)-agathic acid from andrographolides via a regioselective Barton–McCombie reaction. The authors also reported its ^1^H and ^13^CNMR data as well as the negative optical rotation ([α]^20^_D_—55.2, MeOH) [36].

**Figure 3:**
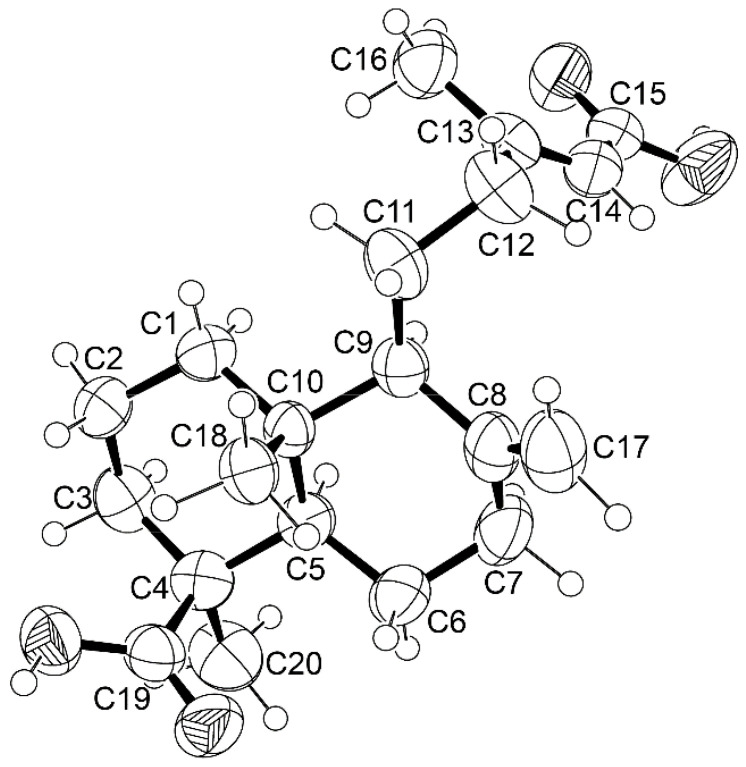
Ortep view of **2a**.

Since **2a** is dextrorotatory, with [α]^20^_D_ + 56.6 (MeOH), we concluded that **2a** is (+)-agathic acid. However, the final proof of the structure of **2a** was provided by X-ray crystallographic analysis, and the ortep view of **2a** is shown in Figure 3.

Since the X-ray crystal structure was obtained from the X-ray diffractometer equipped with the CuKα radiation, the absolute configurations at C-4, C-5, C-9 and C-10 were unequivocally established as 4*S*, 5*R*, 9*S*, 10*R.* To the best of our knowledge, this is the first X-ray crystal structure of (+)-agathic acid.

Analysis of the 1D and 2D NMR spectral data (Table S2, Figures S12–S16) of **2b** revealed its identity as hypoxyterpenoid A, a labdane diterpene previously isolated from the culture extract of a mangrove-derived fungus, *Hypoxylon* sp. (Hsl2–6), which was isolated from the branches of *Bruguiera gymnorrhiza*, collected in Beilun River Mouth, Fangchenggang City, Guangxi Province, China [37]. The position of the hydroxyl group at C-2 was confirmed as α by a NOESY correlation from H-2β to Me-18 and not to Me-19 (Figure S17).

**Figure 4:**
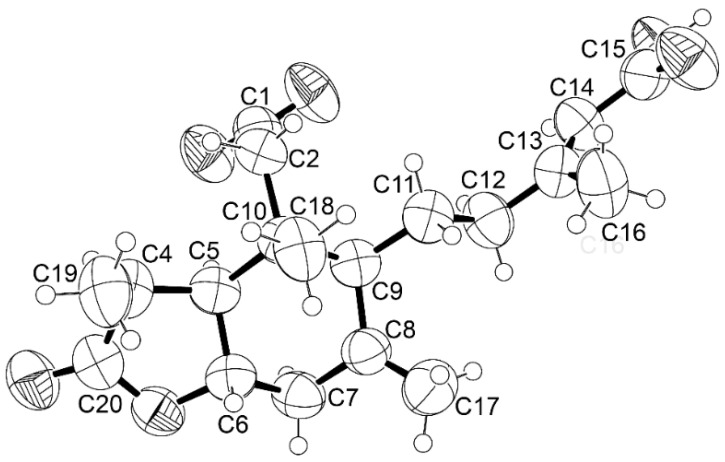
Ortep view of **3**.

Compound **3** was identified as penioxalicin, a 3-*nor*-2,3-seco-labdane-type diterpene on the basis of its 1D and 2D NMR data (Table S3, Figures S18–S22), the optical rotation, and the X-ray crystal structure, whose ortep view is shown in Figure 4. Unfortunately, the value of the Flack parameter we obtained was statistically insignificant; therefore, this specific dataset cannot independently determine the absolute stereochemistry of the compound. However, based on the biogenetic consideration and the co-occurrence of **2a** and **3**, the absolute configurations of the stereogenic carbons in **3** are proposed as 4*S*, 5*S*, 6*S*, 9*S*, 10*R*. Penioxalicin was previously isolated from a culture extract of the fungus *P. oxalicum* TW01-1, obtained from a soil of Xitou mountain in Taiwan, and its X-ray crystal was obtained (the crystal structure obtained through CCDC 1063825 showed the Flack parameter 0.11 (17)), allowing us to determine the absolute configurations of their stereogenic carbons as *4S*, *5S*, *6S*, *9S*, *10R* [38].

Compound **4** was identified as 5-carboxyphthalide based on its ^1^H and ^13^C NMR spectral data (Table S4, Figures S23–S27). This compound was previously reported from a culture extract of the mangrove-derived fungus, *P. aculeatum* (No. 9EB) [39].

**Figure 5:**
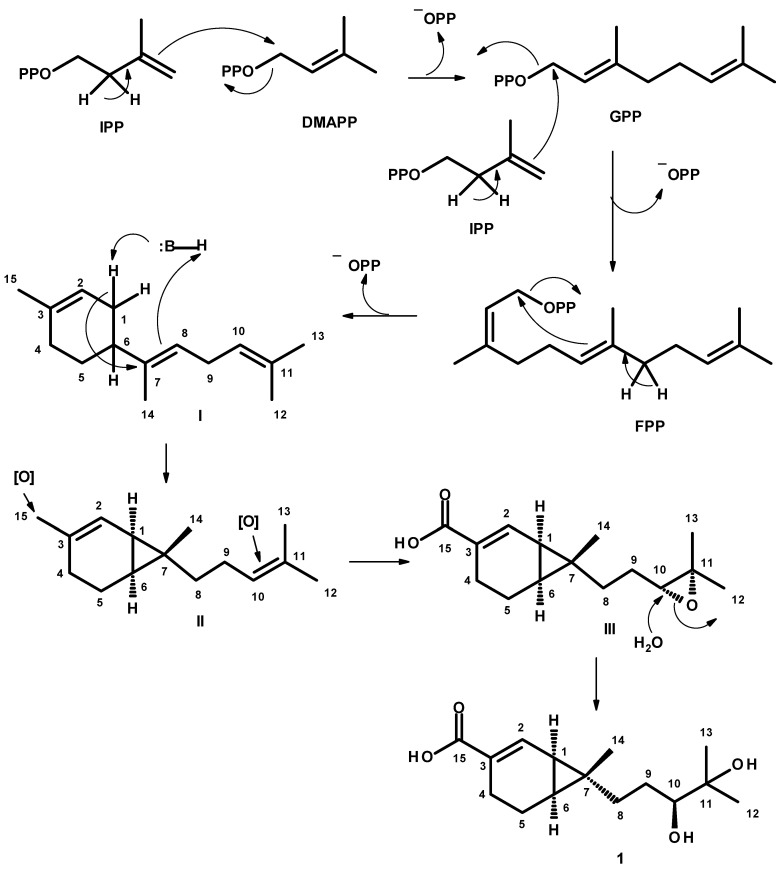
Plausible biogenetic pathway for verruculosic acid (**1**).

Compound **1**, a bicyclic sesquiterpene having a cyclohexene ring fused with a cyclopropane ring, can be hypothesized to derive from a linear sesquiterpene, farnesyl pyrophosphate (FPP). Figure 5 shows the plausible biogenetic pathway for the formation of **1**. The biogenetic pathway leading to **1** starts with a condensation of dimethylallyl pyrophosphate (DMAPP) with isopentenyl pyrophosphate (IPP) to form geranyl pyrophosphate (GPP). Condensation of GPP with another IPP unit gives rise to FPP. Cyclization of FPP by nucleophilic substitution leads to a formation of a methylcyclohexene ring in the intermediate **I**. Basic-catalyzed nucleophilic addition of C-1 to C-7 in **I** leads to a formation of a cyclopropane ring, rendering a 3, 7-dimethylbicyclo [4.1.0]hept-2-ene in **II**. Oxidation of Me-15 and epoxidation of the double bond between C-10 and C-11 result in a formation of **III**. Hydrolysis of the epoxide on C-10 and C-11 gives rise to a *vic*-diol in **1**.

**Figure 6:**
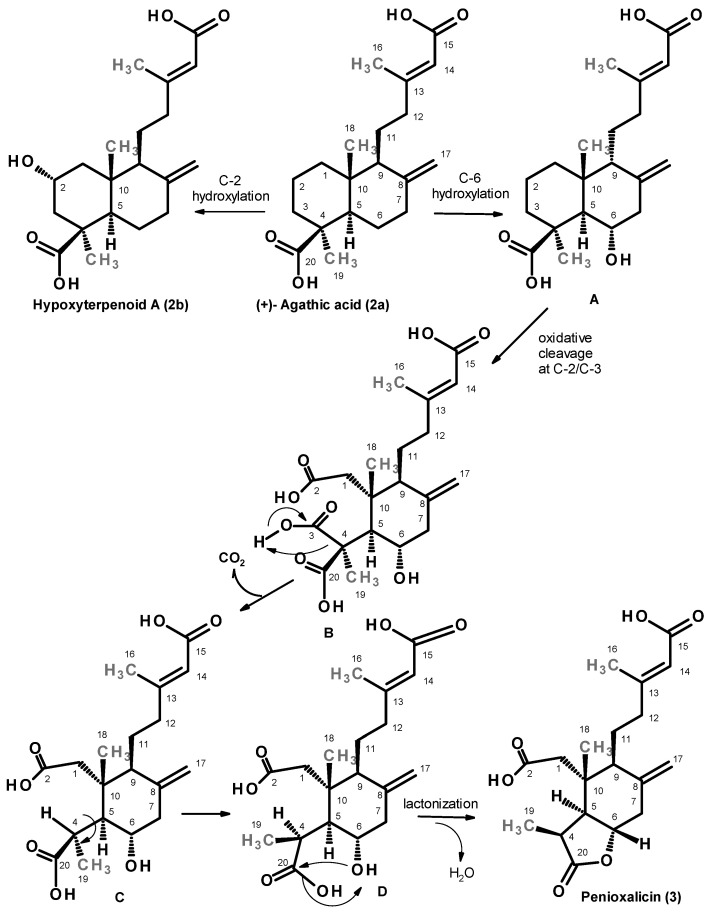
Plausible biogenetic pathway to hypoxyterpenoid A (**2b**) and penioxalicin (**3**) from (+)-agathic acid (**2a**).

Hypoxyterpenoid A (**2b**) and penioxalicin (**3**) can be visualized as products of structural modification of (+)-agathic acid (**2a**). Stereospecific (enzymatic) hydroxylation at C-2 of **2a** yields **2b**. On the other hand, stereospecific (enzymatic) hydroxylation at C-6 of **2a** gives us the intermediate **A**. Oxidative cleavage of the C1-C2 bond in **A** gives rise to a tricarboxylic acid **B** which, after decarboxylation of one carboxyl group on C-4, yields the intermediate **C**. Rotation around the C4–C5 bond in **C** gives rise to the intermediate **D** which, after lactonization by nucleophilic substitution of the carboxyl group on C-4 by the hydroxyl group on C-6, resulted in a formation of **3** (Figure 6).

Although several plant-derived labdane, *nor*-labdane diterpenes [40,41,42,43,44] and sesquiterpenes [45,46] exhibited inhibitory activity against LPS-stimulated NO production in macrophages (BV-2 microglia cells/RAW 264.7 cells), a number of sesquiterpenes and diterpenes from marine-derived fungi have also been reported to exhibit anti-inflammatory activity through NO inhibition in both LPS-induced BV2 cells and LPS-induced RAW 264.7 cells [47]. For this reason, **1**–**4** were tested for their in vitro inhibitory activity against LPS-induced NO production in RAW264.7 macrophages. The IC_50_ values of **1**–**4** are shown in Table 2. Verruculosic acid (**1**), a fused bicyclic sesquiterpene acid, exhibited weaker NO inhibitory activity (IC_50_ = 242.42 µM) than the positive control, diclofenac sodium (IC_50_ = 197.50 µM). Interestingly, among the three labdane diterpenes, (+)-agathic acid (**2a**) showed a slightly weaker inhibitory activity (IC_50_ = 273.44 µM) than **1**. However, the presence of the 2α-hydroxyl group in hypoxyterpenoid A (**2b**) resulted in more than two-fold increase in activity, with IC_50_ = 121.95 µM, when compared to **2a**. On the contrary, the cleavage of ring A of the decahydronaphthalene ring system of the labdane skeleton resulted in a dramatic decrease in activity since the IC_50_ of penioxalicin (**3**) is nearly 3-fold higher than that of **2b**. Compound **4** showed very weak NO inhibitory activity. Interestingly, when compared with the labdane diterpenes, isolated from the methanol extract of the rhizome of *Hedychium coronarium* (family Zingiberaceae), whose side chains of the decahydronaphthalene ring system of the labdane skeleton contain lactone, conjugated lactone, hemiacetal lactone and furan, were much more active, with IC_50_ values ranging from 16 to 97 µM (L-NMMA was used as a positive control), than the side chain with a carboxylic group in **2a** and **2b**. Moreover, the authors found that the inhibition of the NO production by labdane diterpenes from *H. coronarium* was due to their inhibitory activity against the induction of iNOS in LPS-activated macrophages [43]. Dong et al. [44] have found that callicarpaolide, a 3,4-seco-labdane diterpenoid, isolated from *Callicarpa nudiflora* (family Verbenaceae), at a concentration of 50 µM, showed 41.42% of the inhibition rate of the NO production in LPS-activated RAW 264.7 cells (Z,Z’-6,6′,7, 3′α-diligustilide was used as a positive control). However, the authors did not determine the IC_50_ value of the compound to allow a comparison of its potency with labdane diterpenes.

Cell viability assay showed that **1**–**4** had no cytotoxicity against RAW264.7 macrophages at concentrations where cell viability remained above 70% (Table 2).

Compounds **1**–**4** were also tested for antibacterial activity against two Gram-positive (*Staphylococcus aureus* ATCC 29213 and *Enterococcus faecalis* ATCC 29212) and two Gram-negative (*Escherichia coli* ATCC 25922 and *Pseudomonas aeruginosa* ATCC 27853) reference strains, as well as three multidrug-resistant strains, *viz*. an extended-spectrum-lactamase (ESBL)-producing *E. coli* (clinical isolate SA/2), a methicillin-resistant *S. aureus* (MRSA, environmental isolate *S. aureus* 74/24) [48] and a vancomycin-resistant Enterococcus (VRE, environmental isolate *E. faecalis* B3/101) by the Kirby–Bauer method, according to CLSI recommendations [49]. However, none of the tested compounds showed antibacterial activity against all the tested strains.

## 3. Experimental Sections

### 3.1. General Experimental Procedures

The melting points were determined on a Stuart Melting Point Apparatus SMP3 (Bibby Sterilin, Stone, Staffordshire, UK) and were not corrected. Optical rotations were measured on an ADP410 Polarimeter (Bellingham + Stanley Ltd., TunbridgeWells, Kent, UK). ^1^H and ^13^C NMR spectra were recorded at ambient temperature on a Bruker AMC instrument (Bruker Biosciences Corporation, Billerica, MA, USA) operating at 300 or 500 and 75 or 125 MHz, respectively. High-resolution mass spectra were measured with a Waters Xevo QToF mass spectrometer (Waters Corporations, Milford, MA, USA) coupled to a Waters Aquity UPLC system. A Merck (Darmstadt, Germany) silica gel GF254 was used for preparative TLC, and a Merck Si gel 60 (0.2–0.5 mm) (Merck, Darmstadt, Germany) was used for column chromatography. LiChroprep silica gel (Merck KGaA, Darmstadt, Germany) and Sephadex LH 20 (Healthcare Bio-Sciences AB, Uppsala, Sweden), were used for column chromatography.

### 3.2. Fungal Material

The fungus was isolated from the leaves of a sea grass, *Enhalus acoroides* (Family Hydrocharitaceae), which was collected by scuba diving at a depth of 5 m, from the coral reef at Hong Island (8.079081437628979, 98.6811438121823), Krabi province, Thailand, in October 2020. The sea grass was washed with sterilized seawater three times, and then dried on a sterile filter paper under sterile conditions. The leaves of the sea grass were cut into small pieces (5 × 5 mm), four of which were placed on Petri dish plates containing 15 mL potato dextrose agar (PDA) medium mixed with 300 mg/L of streptomycin sulfate, and incubated at room temperature for 7 days. The hyphal tips emerging from the sea grass pieces of leaves were individually transferred onto a PDA slant and maintained as pure cultures at Kasetsart University Fungal Collection, Department of Plant Pathology, Faculty of Agriculture, Kasetsart University, Bangkok, Thailand. The fungal strain KUFA1509 was identified as *Penicillium verruculosum*, based on morphological characteristics. This identification was confirmed by molecular techniques using ITS primers. DNA was extracted from young mycelia according to the method described by Murray and Thompson [50]. The universal primer pairs ITS1 and ITS4 were used for ITS gene amplification [51]. PCR reactions were conducted on a Thermal Cycler, and the amplification process consisted of initial denaturation at 95 °C for 5 min, 34 cycles at 95 °C for 1 min (denaturation), at 55 °C for 1 min (annealing) and at 72 °C for 1.5 min (extension), followed by final extension at 72 °C for 10 min. PCR products were examined by agarose gel electrophoresis (1% agarose with 1 × TBE buffer) and visualized under the UV light after staining. DNA sequencing analyses were conducted using the dideoxyribonucleotide chain termination method [52] by Macrogen Inc. (Seoul, Korea). The DNA sequences were edited using FinchTV 1.4.0 and submitted into the BLAST program (https://blast.ncbi.nlm.nih.gov/, accessed on 19 June 2024) for alignment and compared with that of fungal species in the NCBI database (http://www.ncbi.nlm.nih.gov/, accessed on 19 June 2024). Its gene sequences were deposited in GenBank with the accession number PQ490461.

### 3.3. Extraction and Isolation

*Penicillium verruculosum* KUFA1509 was cultured for one week at 28 °C in five Petri dishes (i.d. 90 mm) containing 20 mL of PDA per dish. The mycelial plugs (5 mm in diameter) were transferred to 500 mL Erlenmeyer flasks containing 200 mL of potato dextrose broth, and incubated on a rotary shaker at 120 rpm at 28 °C for one week at room temperature to prepare a mycelial suspension.

Fifty 1000 mL Erlenmeyer flasks, each containing 300 g cooked rice, were autoclaved at 121 °C for 15 min. After cooling to room temperature, 20 mL of a mycelial suspension of the fungus was inoculated per flask and incubated at 28 °C for 30 days, after which 500 mL of EtOAc was added to each flask and macerated for 7 days. The suspension was then filtered with Whatman No. 1 filter paper to give us the organic solutions, which were combined and then evaporated under reduced pressure to furnish 60 g of the crude EtOAc extract. The crude EtOAc extract was then dissolved in CHCl_3_ (500 mL), washed with H_2_O (3 × 500 mL) and dried over anhydrous Na_2_SO_4_, and filtered with Whatman No. 1 filter paper. The CHCl_3_ solution was evaporated under reduced pressure to obtain 40 g of a crude CHCl_3_ extract, which was applied over a silica gel column (390 g), and eluted with mixtures of petrol-CHCl_3_ and CHCl_3_-Me_2_CO, wherein 250 mL fractions (frs) were collected as follows: frs 1–116 (petrol-CHCl_3_, 1:1), 117–174 (petrol-CHCl_3_, 3:7), 175–242 (petrol-CHCl_3_, 1:9), 243–361 (CHCl_3_-Me_2_CO, 9:1), 362–478 (CHCl_3_-Me_2_CO, 7:3), 479–545 (CHCl_3_-Me_2_CO, 1:1). Frs 310–323 were combined (767 mg) and precipitated in CH_3_CN to give us 38.2 mg of colorless crystals of **2a**. Frs 338–356 were combined (547.5 mg) and applied over a Sephadex LH-20 column (15 g), and eluted with MeOH, wherein 16 subfractions (sfrs) of 1 mL were collected. Sfrs 7–11 were combined (530 mg) and applied over another Sephadex LH-20 column (5 g), and eluted with CHCl_3_, wherein 16 sub-subfractions (ssfrs) of 1 mL were collected. Ssfrs 12–16 were combined (367.6 mg) and purified by preparative TLC (sílica gel G_254_, CHCl_3_: Me_2_CO: HCO_2_H; 80:20:0.1) to give us 13.3 mg of **1**. Frs 365–374 were combined (672 mg) and precipitated in CHCl_3_ to give us 51 mg of **2b**. Frs 375–398 were combined (940 mg) and precipitated in CHCl_3_ to give us 70.3 mg of **3**. The mother liquor was applied over a Sephadex LH-20 column (15 g), and eluted with MeOH, wherein 15 sfrs of 2 mL were collected. Sfrs 12–13 were combined to give us 9.8 mg of **4**.

#### Verruculosic Acid (**1**)

Colorless crystals. Mp. 80–82 °C. [α${]}_{D}^{20}$-160 (*c* 0.05, MeOH); for ^1^H and ^13^C spectroscopic data (DMSO-*d6*, 300 and 75 MHz), see Table 1; and (+)-HRESIMS *m/z* 251.1654 [M-H_2_O+H]^+^ (calculated for C_15_H_23_O_3_, 251.1647).

### 3.4. X-Ray Crystal Structures

Diffraction data were collected with a Rigaku/Oxford Diffraction Gemini PX Ultra single-crystal X-ray diffractometer equipped with CuK_α_ radiation (λ = 1.54184 Å) at 291 K. The structures were solved by direct methods, using SHELXS-97 (version 97-2), and refined with SHELXL-97 [53]. Carbon and oxygen were refined anisotropically. Hydrogen atoms were either placed at their idealized positions using appropriate HFIX instructions in SHELXL, and included in subsequent refinement cycles, or were directly found from difference Fourier maps and were refined freely with isotropic displacement parameters. Full details of the data collection and refinement and tables of atomic coordinates, bond lengths and angles, and torsion angles are deposited in the Cambridge Crystallographic Data Centre.

#### 3.4.1. X-Ray Crystal Structure of **1**

Crystals were monoclinic, space group P2_1_, cell volume 1533.51(8) Å^3^, unit cell dimensions *a* = 6.53248(18) Å, *b* = 23.6959(7) Å and *c* = 10.2335(3) Å, and angle *β* = 104.516(3)° (uncertainties in parentheses). The refinement converged to *R* (all data) = 6.87% and *wR*_2_ (all data) = 12.08% (flack parameter (x) of 0.0(3)). CCDC 2538472.

#### 3.4.2. X-Ray Crystal Structure of **2a**

Crystals were orthorhombic, space group P2_1_2_1_2_1_, cell volume 1896.60(11) Å^3^ and unit cell dimensions *a* = 12.0993(3) Å, *b* = 12.4811(5) Å and *c* = 12.5592(4) Å. The refinement converged to *R* (all data) = 5.83% and *wR*_2_ (all data) = 9.91% (flack *x* parameter 0.1(5)). CCDC 2538243.

#### 3.4.3. X-Ray Crystal Structure of **3**

Crystals were monoclinic, space group C2, cell volume 1878.7(4) Å^3^ and unit cell dimensions *a* = 28.141(4) Å, *b* = 6.8096(6) Å and *c* = 9.8796(13) Å, and angle *β* = 97.084(11)°. The refinement converged to *R* (all data) = 10.57% and *wR*_2_ (all data) = 30.35%. CCDC 2538253.

### 3.5. Nitric Oxide (NO) Inhibitory Activity and Cytotoxicity of **1–4** Against RAW 264.7 Macrophages

The inhibitory effect of **1**–**4** on nitric oxide (NO) production was evaluated in LPS (Sigma-Aldrich, St. Louis, MO, USA)-activated murine macrophage RAW 264.7 cell lines (ATCC^®^, Lot: 70012232, Biomedia Co., Ltd., Bangkok, Thailand), according to the previously described method with some modifications [54]. RAW 264.7 cells were cultured in Dulbecco’s Modified Eagle’s Medium (DMEM) (Gibco^®^, Life Technologies Corporation, New York, NY, USA) supplemented with 10% fetal bovine serum (Gibco^®^, Life Technologies Limited, Paisley, UK) and 1% antibiotic–antimycotic solution (10 × 10^3^ units/mL of penicillin, 10 mg/mL of streptomycin, and 25 µg/mL of amphotericin B (Gibco^®^, Life Technologies Corporation, New York, NY, USA). The cells were maintained at 37 °C in an incubator with 5% CO_2_ and 95% humidity. For the NO inhibitory assay, the RAW 264.7 cells (1 × 10^5^ cells/well) in DMEM were seeded into 96-well plates containing 100 μL of culture medium and incubated for 24 h. Subsequently, the medium was replaced with 100 μL of the fresh medium containing 2 μg/mL of LPS. Then, the test samples, at final concentrations ranging from 100, 50, 10 and 1 µg/mL (except for compound **4**, which was tested at final concentrations of 150, 75, 50, and 10 μg/mL.) were added to each well. A total of 0.5% DMSO (final concentration) was used in the solvent control wells (except for compound **4**, where 0.75% DMSO final concentration was used). After 24 h of incubation, 100 μL of supernatant was transferred to new 96-well plates, and 100 μL of Griess reagent (1% sulfanilamide in 0.1% *N*-(1-naphtyl)ethylenediamine dihydrochloide in 2.5% H_3_PO_4_ solution) was added to each well. The absorbance (Abs) of the samples and the control was measured at 520 nm using a microplate reader (SPECTROstar Nano, BMG LABTECH, Ortenberg, Germany).

The assay was carried out in triplicate, and diclofenac sodium (Thermo Scientific, Shanghai, China), at final concentrations ranging from 100, 50, 10 and 1 µg/mL, was used as a positive control.

The percentage of NO inhibition was determined according to the following equation:

% Inhibition of NO production = [Abs_sample_ − Abs_control_/Abs_control_] × 100

The IC_50_ values were calculated using the GraphPad Prism version 8 (GraphPad Software, Boston, MA, USA) from a dose–response curve, which was generated by plotting the percentage of NO inhibition against the concentrations of the test compound. The experiments were performed using concentrations in µg/mL. After the IC_50_ values were obtained, they were converted from µg/mL to µM.

Cell viability of RAW 264.7 macrophages, under the same treatment conditions, was also assessed using the MTT assay. Briefly, after removal of the culture supernatant, MTT solution at 5 mg/mL was added to each well, and incubated at 37 °C in 5% CO_2_ for 2 h. Then, the supernatant was removed, and 50 μL of DMSO was added to dissolve the formazan product in cells. The absorbance of the formazan solution was measured at 570 nm using a microplate reader. The results were considered valid when the cell viability remained higher than 70% when compared with the control.

### 3.6. Statistical Analysis

NO inhibitory activity assays were performed in triplicate. Values for different parameters were expressed as the mean ± standard deviation (SD). The data were statistically analyzed using one-way analysis of variance (ANOVA), followed by Tukey’s post hoc test. The level of significance was set at *p* < 0.01. Statistical analysis was performed using GraphPad Prism version 8 (GraphPad Software, Boston, MA, USA).

## 4. Conclusions

Although a myriad of pharmacologically relevant compounds have been reported from marine-derived *Penicillium* species, the investigation of secondary metabolites of *P. verruculosum* was very scarce. Intriguingly, among biological and pharmacological activities investigated for compounds isolated from marine-derived *Penicillium* species, only few studies on inhibition of nitric oxide (NO) production have been reported when compared to other pharmacological activities such as anticancer and antimicrobial activities. Investigation of secondary metabolites from the culture of *P. verruculosum* KUFA1059, isolated from the sea grass collected in the Andaman Sea in Southern Thailand, led to the isolation of one unreported bicyclic sesquiterpene acid, which we have named verruculosic acid, and two previously reported labdane diterpenes, one previously described 3-*nor*-2,3-seco-labdane and 5-carboxyphthalide. Although the planar structure of verruculosic acid is the same as that of the previously reported sesquicaranoic acids A and B, X-ray analysis revealed that verruculosic acid is a diastereomer of sesquicaranoic acids A and B. Investigation of the isolated compounds for their capacity to inhibit LPS-induced NO production in RAW 264.7 macrophages revealed interesting activity of the labdane-type diterpenes. Many labdane-type diterpenoids, mostly from plant sources, are widely recognized for their potent anti-inflammatory properties and exhibit significant inhibitory effects against the LPS-induced NO production. Labdane diterpenes normally suppress NO overproduction by decreasing the expression of the inducible nitric oxide synthase (iNOS) enzyme at the transcriptional level, blocking the nuclear factor kappa B (NF-kB) signaling pathway by preventing the phosphorylation and degradation of kB proteins, which stops inflammatory genes from turning on, and inhibiting mitogen-activated protein kinases (MAPK), further reducing the release of other pro-inflammatory molecules. Therefore, in this study, diclofenac sodium, which primarily works as a non-selective cyclooxygenase (COX) inhibitor to block prostaglandin synthesis, but it also inhibits iNOS gene expression at the transcriptional level (often via the NF-κB pathway), was chosen as a positive control. This choice is suitable for searching for anti-inflammatory compounds. Moreover, in this study it was shown that the capacity of inhibition of the LPS-induced NO production is related to the structural features of the labdane scaffold. While hypoxyterpenoid A, the analog of (+)-agathic acid with a 2α-hydroxyl substituent, was found to increase NO inhibitory activity when compared to (+) agathic acid, with IC_50_ value less than that of diclofenac sodium, the 3-*nor*-2,3-seco-labdane, penioxalicin showed a dramatic decrease in activity. These results can lead to the conclusion that the intact decahydronaphthalene ring system is important for activity. However, when compared to previous reports on the effect of inhibitory activities on LPS-induced NO production by labdane diterpenes with different functional groups on the side chain of the decahydronaphthalene ring system of the labdane skeleton, it was found that the free carboxylic acid on the side chain caused a decrease in activity. Therefore, in order to obtain more insight about the structure–activity relationship of the labdane-type diterpenes, more analogs of these compounds, including those with different substituents on the decahydronaphthalene ring system, have to be tested. Nevertheless, this study demonstrates that the labdane skeleton can be considered to be a promising scaffold for the development of anti-inflammatory agents that interfere with NO production.

## Figures and Tables

**Table 1:** ^1^H and ^13^C NMR (DMSO-*d6*, 300 MHz and 75 MHz) and HMBC assignment for verruculosic acid (**1**).

Position	δ_C_, Type	δ_H_ (*J* in Hz)	COSY	HMBC
1	22.9 CH	1.26, m	H-2, H-6	C-3, 7, 8
2	138.9 CH	7.11, brd (5.3)	H-1, 4, 6	C-1, 6, 15
3	127.7 C	-		
4	21.9 CH_2_	1.78, m 2.28, m	H-5	C-2, 3, 5
5	17.2, CH_2_	1.77, m	H-6	
6	25.0, CH	1.18, m	H-1, 5	
7	33.1, C	-		
8	40.5, CH_2_	1.16, m 1.63, m	H-9	C-7
9	28.4, CH_2_	1.21, m 1.66, m	H-8, 10	
10	77.7, CH	3.03, d (8.9)	H_2_-9	C-8
11	72.1, C	-		
12	26.9, CH_3_	1.05, s		C-10, 11, 13
13	24.9, CH_3_	1.00, s		C-10, 11, 12
14	13.5, CH_3_	0.82, s		C-1, 6, 7, 8
15	168.5, CO	-		

**Table 2:** Nitric oxide (NO) inhibitory activity and cytotoxicity of **1**–**4** against RAW264.7 macrophages stimulated by LPS.

Compounds	NO Inhibitory Activity (IC_50_, µM)	% Cell Viability
**1**	242.42 ± 3.37 ^a^	102.15 ± 5.48
**2a**	273.44 ± 4.79 ^b^	104.03 ± 3.44
**2b**	121.95 ± 3.90 ^c^	90.13 ± 2.50
**3**	349.23 ± 9.75 ^d^	93.28± 7.21
**4**	519.79 ± 13.24 ^e^	97.98 ± 4.92
Diclofenac sodium	197.50 ± 3.71	99.56 ± 4.10

The values are expressed as mean ± SD (*n* = 3). Different superscript letters indicate significant differences among groups (one-way ANOVA, followed by Tukey’s post hoc test, *p* < 0.01).

## Data Availability

The original contributions presented in this study are included in the article.

## Supplementary Materials

The following supporting information can be downloaded at: https://www.mdpi.com/article/10.3390/md24060205/s1.
