# Supplementary material for: New Bicyclic Sesquiterpene and Labdane Diterpenes from the Culture Extract of the Sea Grass-Derived Fungus Penicillium verruculosum KUFA1509
Source: Mar Drugs. 2026 Jun 10;24(6):205. doi: 10.3390/md24060205 (PMC13301828; doi:10.3390/md24060205)

Supplementary Materials

New Bicyclic Sesquiterpene and Labdane Diterpenes from the Culture Extract of the Sea Grass-Derived Fungus *Penicillium verruculosum* KUFA1509 §

Diana I. C. Pinho[1, 4], Tida Dethoup[2], Ruchiluk Rattarom[3], Emília Sousa[4], Salar Hafez-Ghoran[5], Artur M. S. Silva[6], Luís Gales[7]*, Anake Kijjoa[1]*

[1] School of Medicine and Biomedical Sciences Abel Salazar (ICBAS) and CIIMAR, Rua de Jorge Viterbo Ferreira, 228, 4050-313 Porto, Portugal; up201706356@edu.ff.up.pt (D.P.)

[2] Department of Plant Pathology, Faculty of Agriculture, Kasetsart University, Bangkok 10240, Thailand; agrtdd@ku.ac.th (T.D.)

[3] Pharmaceutical Chemistry and Natural Product Research Unit, Faculty of Pharmacy, Mahasarakham University, Kantharawichai, Maha Sarakham 44150, Thailand; rujiluk.r@msu.ac.th (R.R.)

[4] Laboratório de Química Orgânica e Farmacêutica, Departamento de Ciências Químicas, Faculdade de Farmácia, Universidade do Porto and CIIMAR, Rua de Jorge Viterbo Ferreira 228, 4050-313 Porto, Portugal; esousa@ff.up.pt (E.S.)

[5] Laboratory for Functional Foods and Human Health, Center for Excellence in Post-Harvest Technologies, North Carolina Agricultural and Technical State University, North Carolina Research Campus, 500 Laureate Way, Kannapolis, North Carolina 28081, United States; S_Hafezghoran@yahoo.com (S.H.G.)

[6] Departamento de Química & QOPNA, Universidade de Aveiro, 3810-193 Aveiro, Portugal; artur.silva@ua.pt (A.M.S.S.)

[7] School of Medicine and Biomedical Sciences Abel Salazar (ICBAS) and Instituto de Biologia Molecular e Celular (i3S-IBMC), Rua de Jorge Viterbo Ferreira, 228, 4050-313 Porto, Portugal; lgales@ibmc.up.pt (L. G.)

*Correspondence: ankijjoa@icbas.up.pt (A.K.); Tel. +351-962712474; and lgales@ibmc.up.pt (L. G.); Tel. +351-918945358

Table S1. ^{1}H and ^{13}C NMR (DMSO-d6, 300 MHz and 75 MHz) and HMBC assignment for Agathic acid (**2a**)

Position	δ_C, type	δ_H (*J* in Hz)	COSY	HMBC
1	39.6, CH_2	2.20, m		
2	20.1, CH_2	1.45, m<br>1.78, m		
3	38.1, CH_2	0.97, dd (13.0, 3.5)<br>2.01, brd (13.0)		
4	43.8, C	-		
5	55.5, CH	1.30, brd (11.5)		C-20
6	26.4, CH_2	1.70, m<br>1.90, m		C-8
7	38.6, CH_2	1.78, m<br>2.35, m		C-8, 17
8	148.2, C	-		
9	55.1, CH	1.57, brd (10.5)	H-17	C-8, 17
10	39.4, C	-		
11	22.7, CH_2	1.65, m		
12	39.0, CH_2	1.06, dd (13.0, 3.5)<br>1.90, m		
13	159.6, C	-		
14	116.4, CH	5.53, d (0.7)	H-16	C-12, 13, 15
15	167.9, CO	-		
16	18.7, CH_3	2.07, d (1.1)	H-14	C-13, 16, 15
17	106.7, CH_2	4.50, s<br>4.86, s	H-9	C-7, 9
18	13.0, CH_3	0.55, s		C-1, C-9
19.	29.1, CH_3	1.12, s		C-3, 4, 5, 20
20	178.8, CO	-		
OH-20	-	11.95, br		

Table S2. ^{1}H and ^{13}C NMR (DMSO-*d6*, 300 MHz and 75 MHz) and HMBC assignment for hypoxyterpenoid A (**2b**)

Position	δ_C, type	δ_H (*J* in Hz)	COSY	HMBC
1	48.4, CH_2	0.88, ddd (12.2, 12.2, 2.3)<br>1.99, m	H-2	C-2, 18, 20
2	63.3, CH	3.88, m	H-1, 3	
3	47.3, CH_2	2.21, m	H-2, 4	
4	44.7, C	-		
5	54.9, CH	1.26, dd (12.1, 2.3)	H-4,	C-6, 10, 18
6	26.0, CH_2	1.71, m<br>1.89, m		
7	38.2, CH_2	1.85, m<br>1.35, m		
8	147.8, C	-		
9	55.1, CH	1.61, d (10.3)		C-8, 10, 17
10	41.3, C	-		
11	21.9, CH_2	1.48, m<br>1.63, m		
12	39.5, CH_2	1.96, m<br>2.21, m		

13	159.6, C	-		
14	116.4, CH	5.55, s	H-9, 16	C-12, 15, 16
15	167.9, CO	-		
16	18.7, CH_3	1.08, d (1.0)		C12, 13, 14
17	107.2, CH_2	4.52, s<br>4.89, s		C-7, 8, 9
18	13.9, CH_3	0.55, s		C-1, 5, 9, 10
19	29.1, CH_3	1.16, s		C-4. 9, 10, 20
20	178.7CO	-		

Table S3. ^{1}H and ^{13}C NMR (DMSO-*d6*, 300 MHz and 75 MHz) and HMBC assignment for penioxalicin (**3**).

Position	δ_C, type	δ_H (*J* in Hz)	COSY	HMBC
1	42.2, CH_2	2.34, d (14.2)<br>2.47, d (14.2)		C-2
2	173.0, CO	-		
4	38.7, CH	2.79, dq (7.4, 7.8)	H-5, 19	C-6, 19, 20
5	52.8, CH	2.42, dd (11.4, 7.8)	H-4, 6	C-6, 9, 12, 18
6	76.9, CH	4.38, ddd (11.4, 11.4, 4.7)	H-5, 7	
7	42.6, CH_2	2.15, dd (11.2, 11.2)<br>2.90, dd (11.2, 4.7)	H-6, 17	C-6, 8
8	142.6, C	-		
9	50.8, CH	2.08, d (14.2)	_H-19	
10	40.4, C	-		
11	22.7, CH_2	1.50, m<br>1.70, m	H-12	
12	39.6, CH_2	1.89, m<br>2.19, m	H-11	
13	159.4, C	-		
14	116.3, CH	5.58, d, (0.7)	H-16	C-12, 15, 16
15	!67.0, CO	-		
16	18. 8, CH_3	2.10, d (1.1)	H-14	C-12, 13, 14, 15
17	113.9, CH_2	4.85, s<br>5.20, s	H-7, 9	C-7, 9
18	17.8, CH_3	0.79, s		C-2, 9, 10
19	12.1, CH_3	1.19, d (7.6)	H-4	C-4, 5
20	179.4, CO	-		
OH-2	-	12.10, br		

Table S4. ^{1}H and ^{13}C NMR (DMSO-*d6*, 300 MHz and 75 MHz) and HMBC assignment for **5-**carboxyphthalide (**4**).

Position	δ_C, type	δ_H (*J* in Hz)	COSY	HMBC
1	170.9 CO	-		
2	128.9 C	-		
3	125.6 CH	7.95 dd (8.0, 0.7)	H-4	C-1, 5, 7
4	130.2 CH	8.11 dd (8.0, 1.9)	H-3	C-2, 6, 9
5	136.5 C	-		
6	124.5 CH	8.22 dd (1.9, 0.8)		C-2, 4, 9
7	148.1 C	-		
8	70.6 CH_2	5.48s		C-1, 2, 5, 7
9	167.0 CO	-		

Figure S1. ^{1}H NMR spectrum of **1** (DMSO$_{d6}$, 300 MHz).

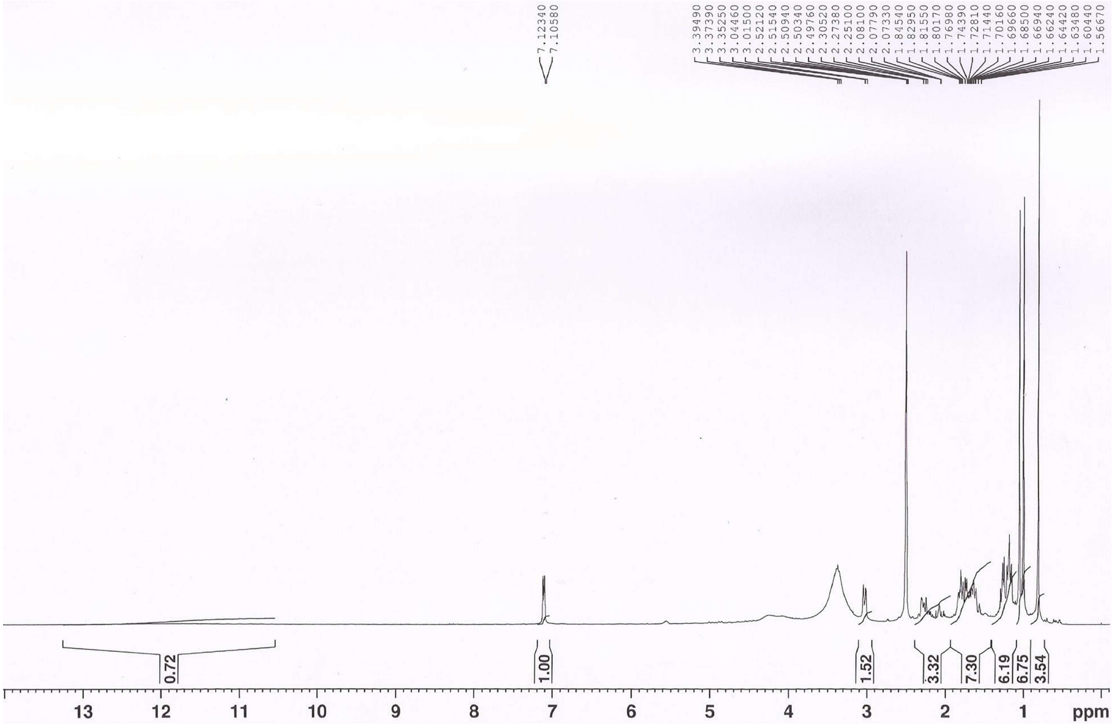

Figure S2. ^{13}C NMR spectrum of **1** (DMSO$_{d6}$, 75 MHz).

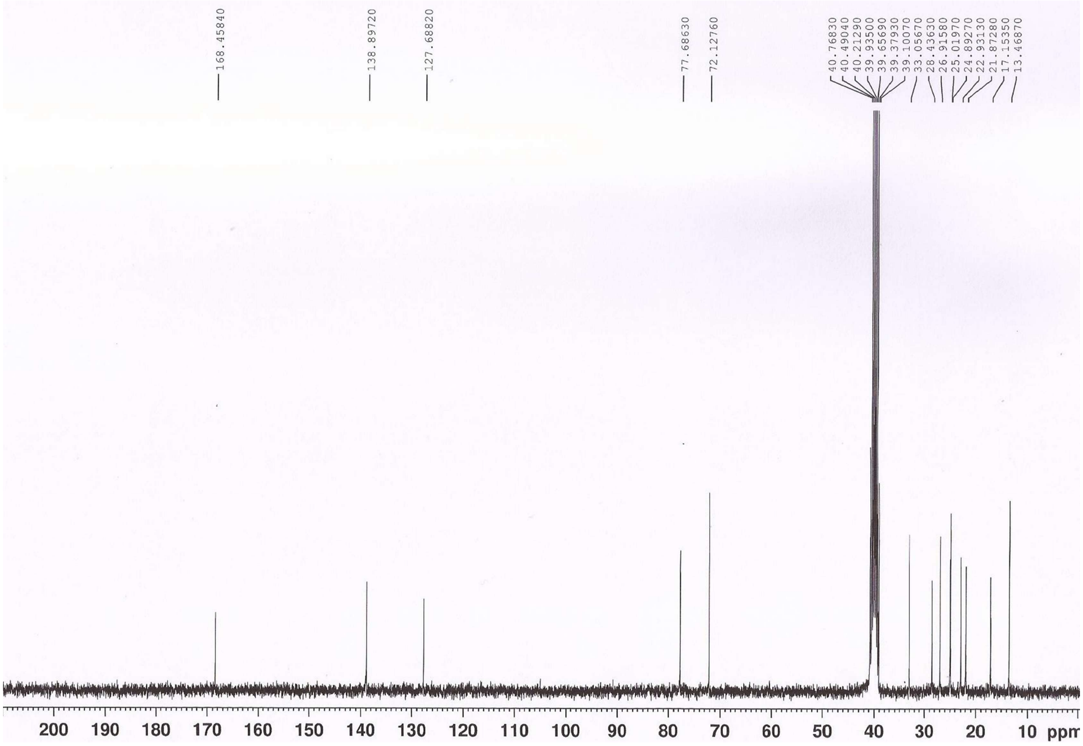

Figure S3. COSY spectrum of **1** (DMSO$_{d6}$, 300 MHz).

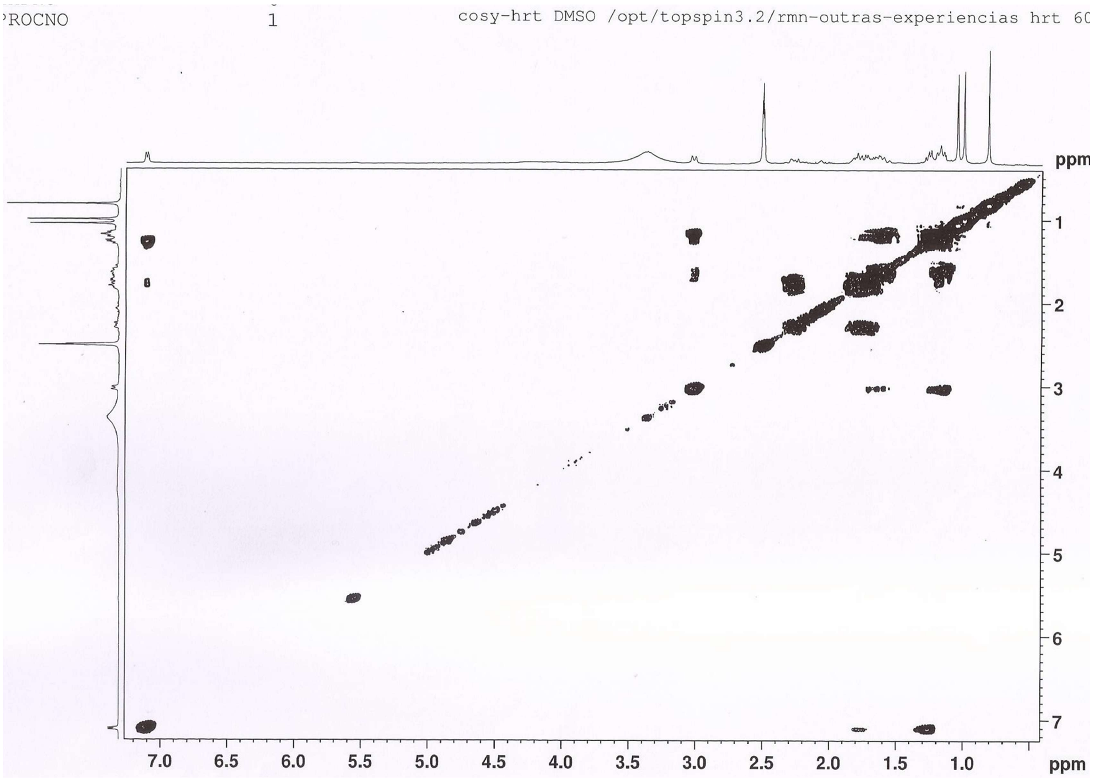

Figure S4. HSQC spectrum of **1** (DMSO$_{d6}$, 300 MHz).

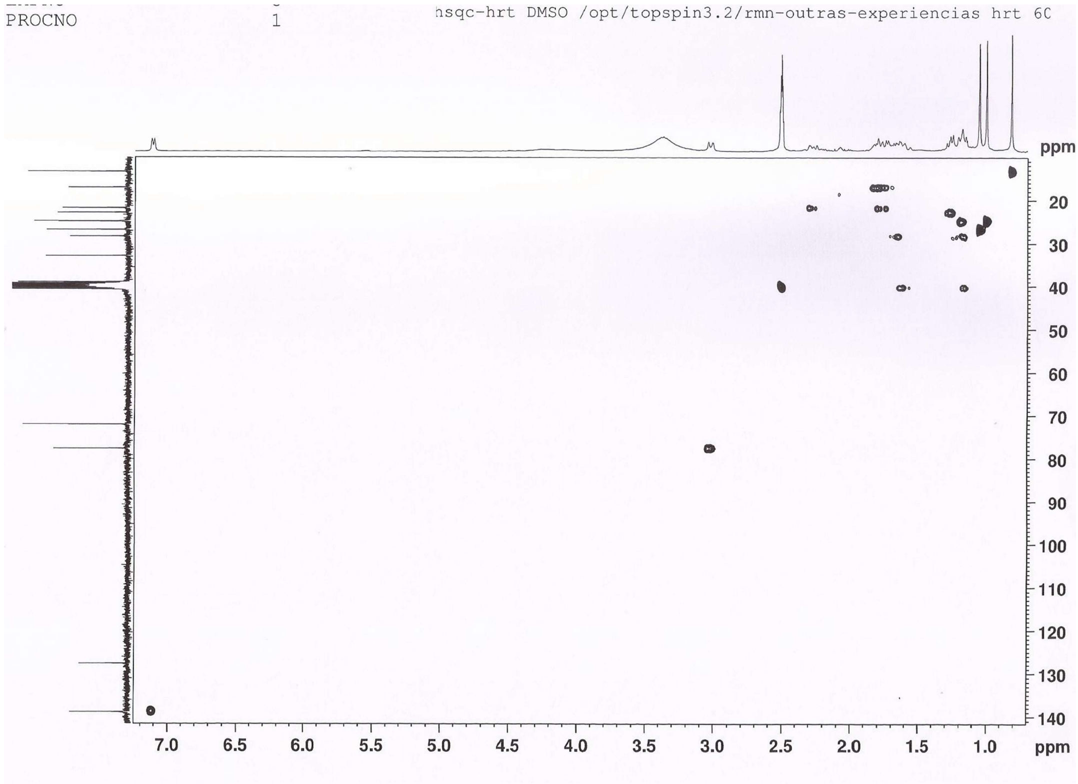

Figure S5. HMBC spectrum of **1** ($DMSO_{d6}$, 300 MHz).

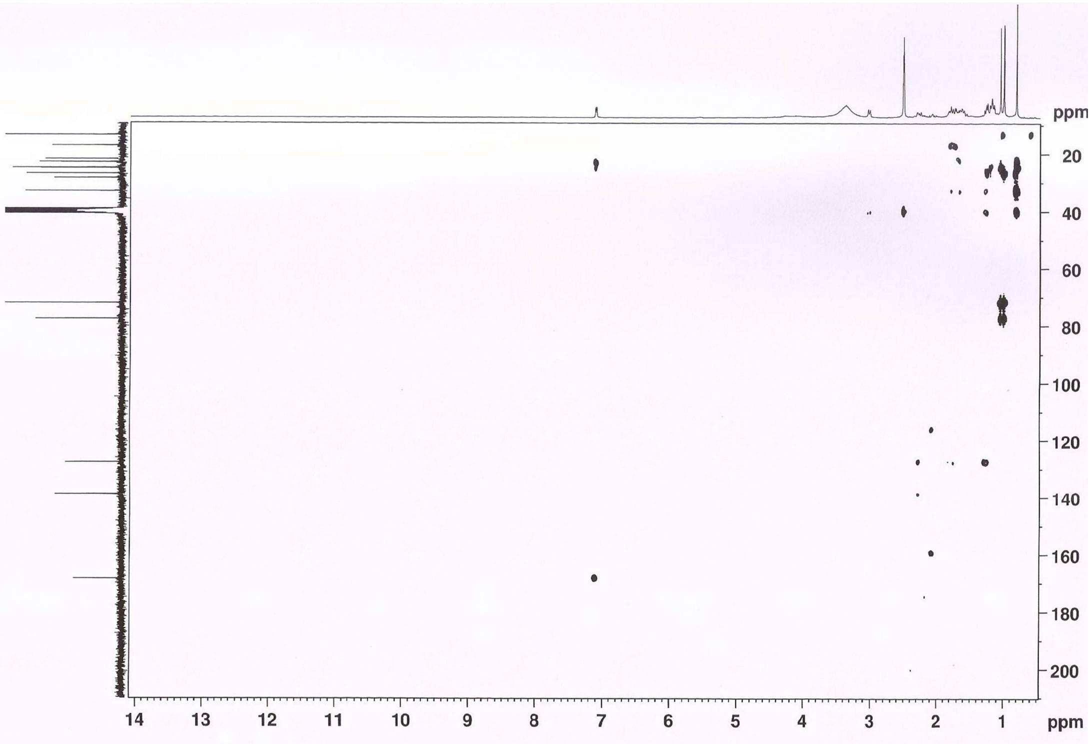

Figure S6. ROESY spectrum of **1** ($DMSO_{d6}$, 300 MHz).

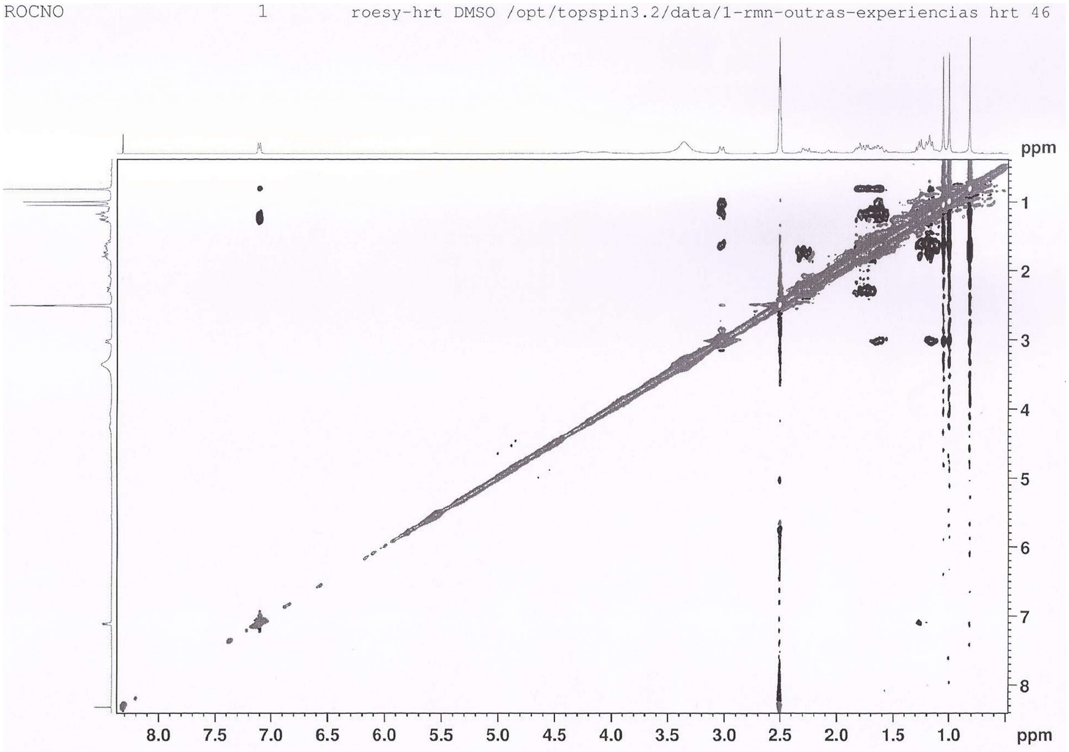

Figure S7. ^{1}H spectrum of **2a** (DMSO$_{d6}$, 300 MHz).

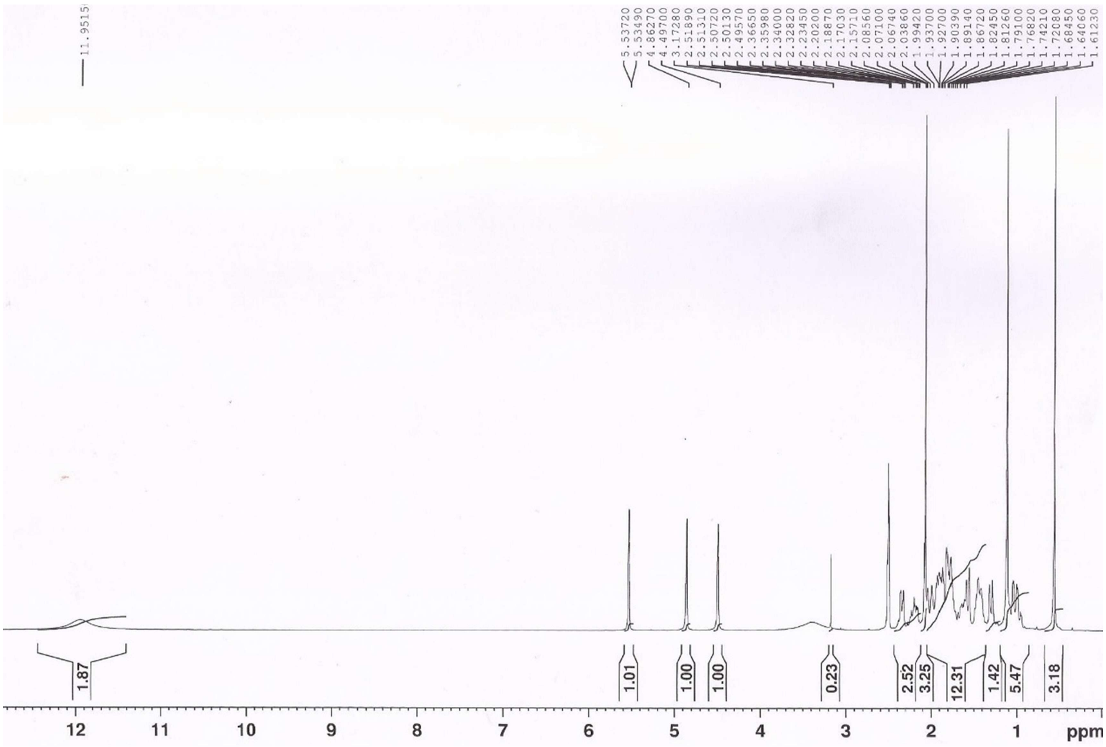

Figure S8. ^{13}C spectrum of **2a** (DMSO$_{d6}$, 75 MHz).

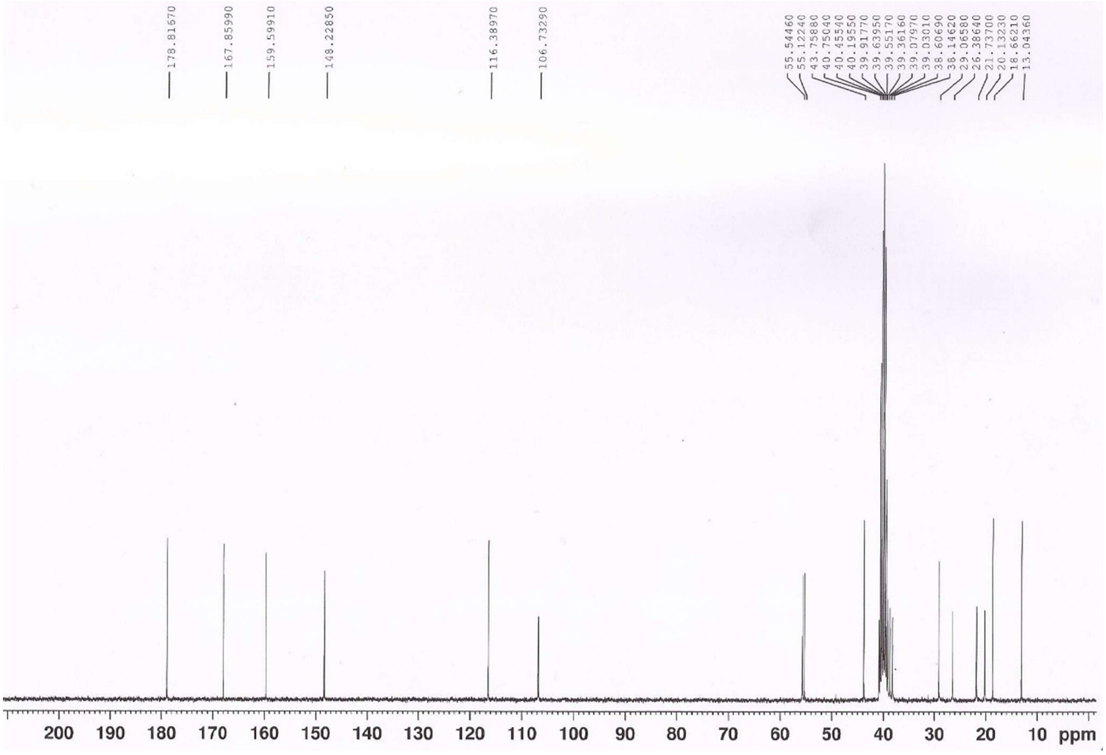

Figure S9. COSY spectrum of **2a** ($DMSO_{d6}$, 300 MHz).

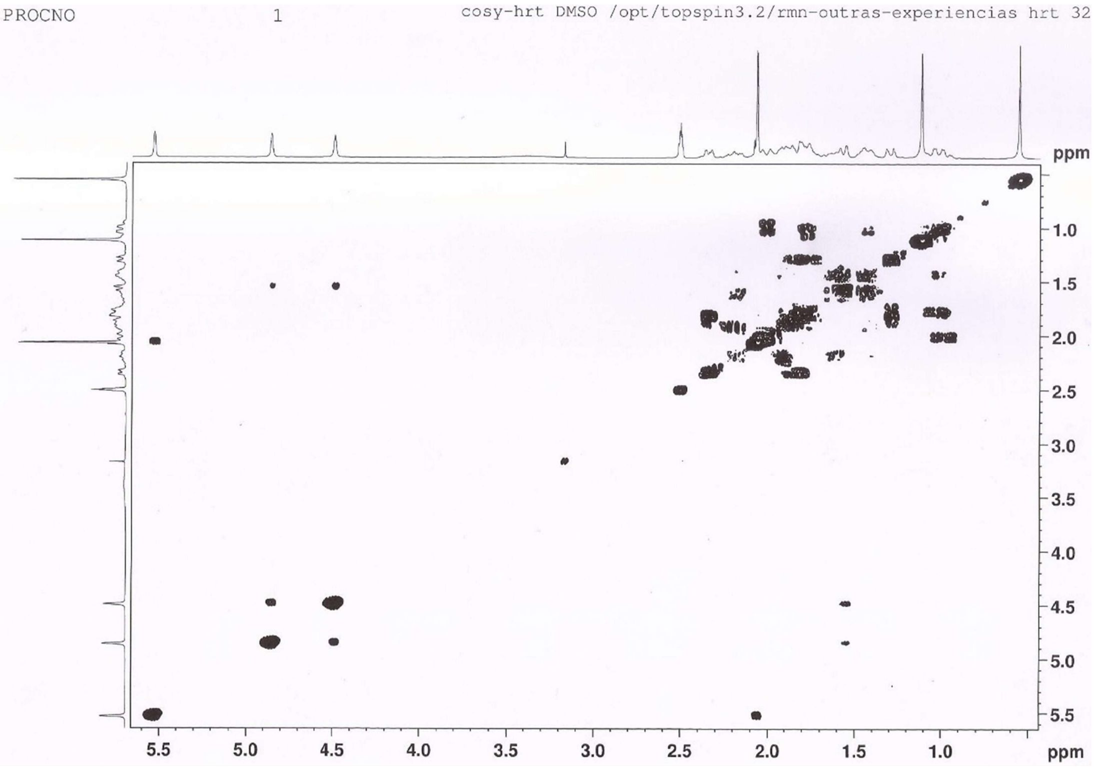

Figure S10. HSQC spectrum of **2a** ($DMSO_{d6}$, 300 MHz).

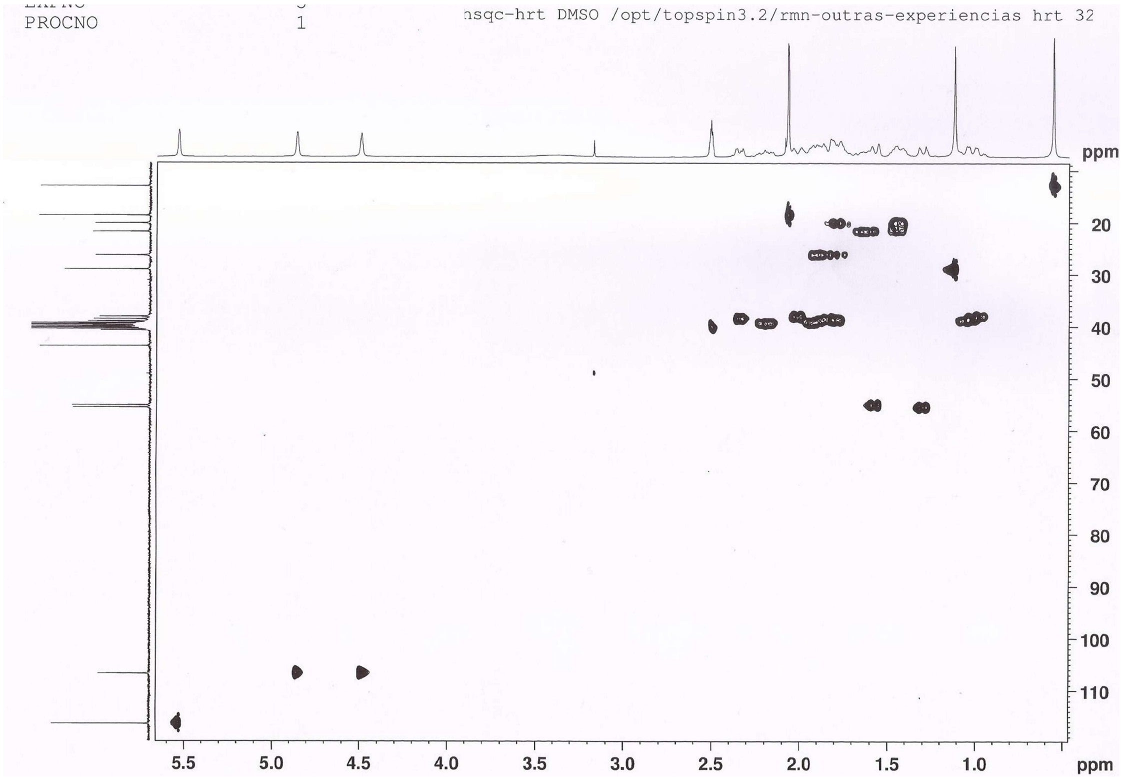

Figure S11. HMBC spectrum of **2a** ($DMSO_{d6}$, 300 MHz).

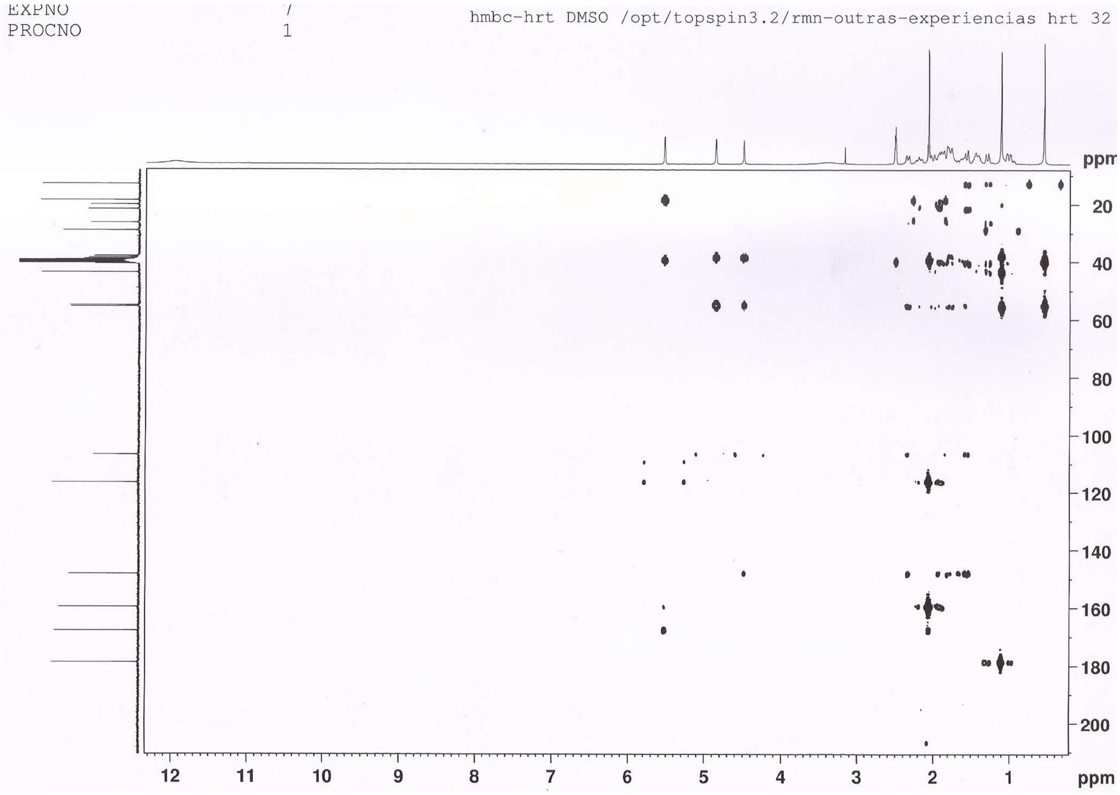

Figure S12. ^{1}H NMR spectrum of **2b** ($DMSO_{d6}$, 300 MHz).

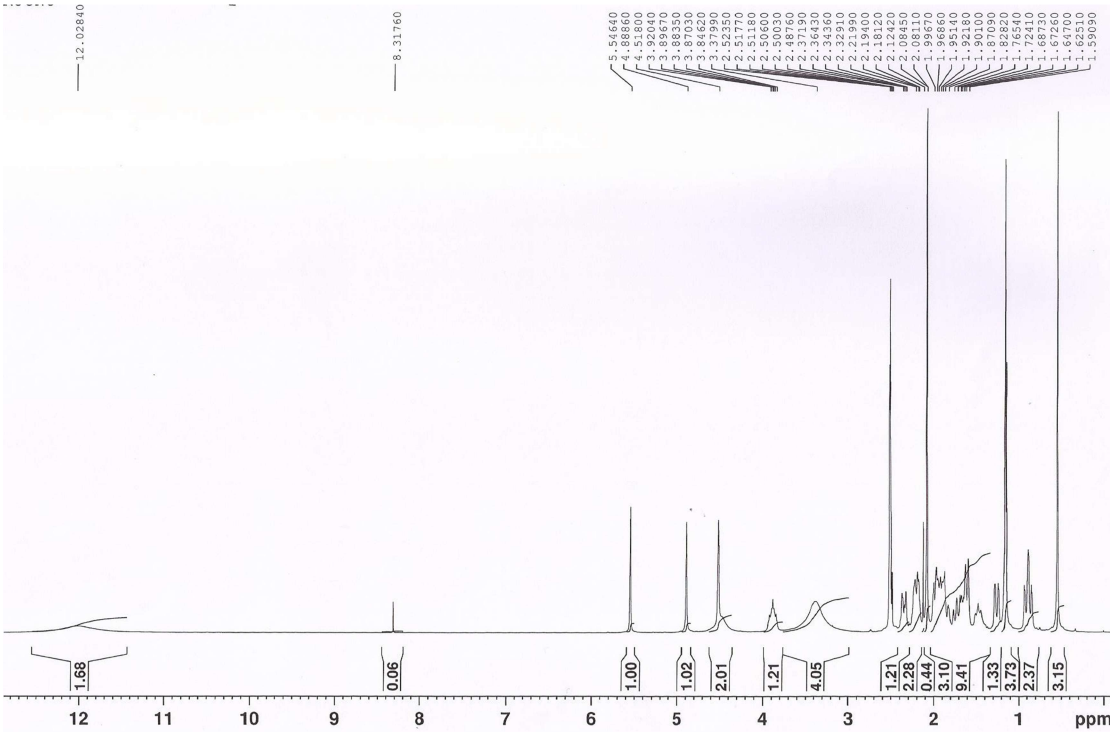

Figure S13. ^{13}C NMR spectrum of **2b** (DMSO$_{d6}$, 75 MHz).

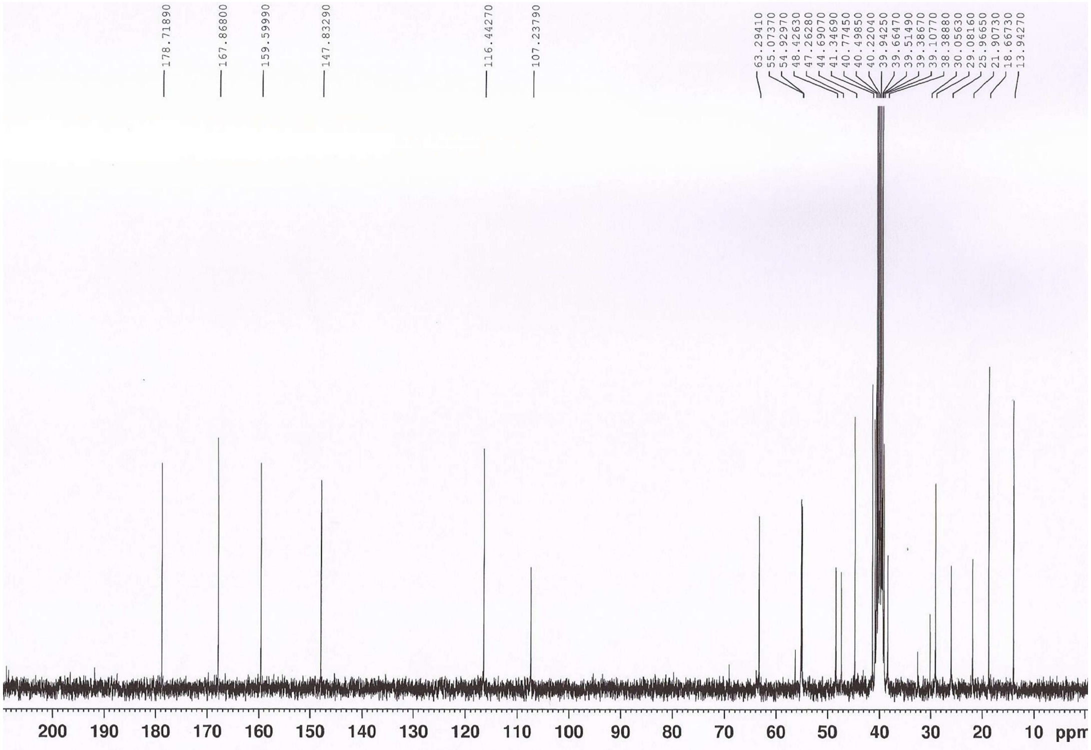

Figure S14. COSY spectrum of **2b** (DMSO$_{d6}$, 300 MHz).

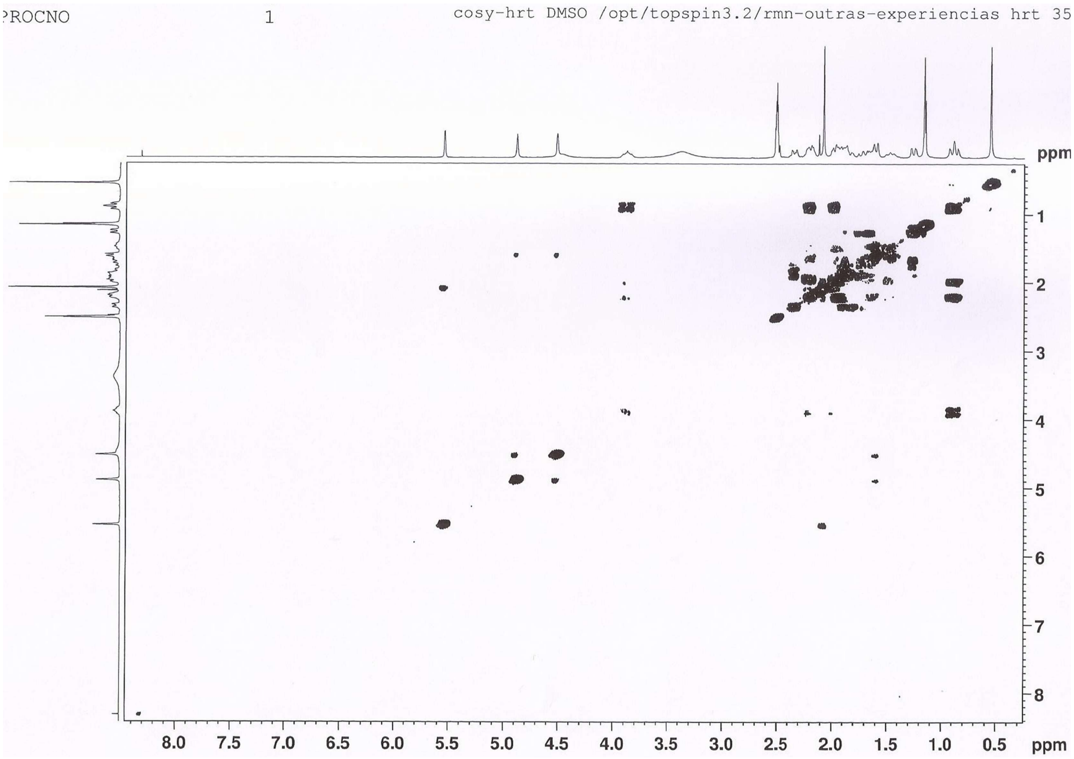

Figure S15. HSQC spectrum of **2b** (DMSOd6, 300 MHz).

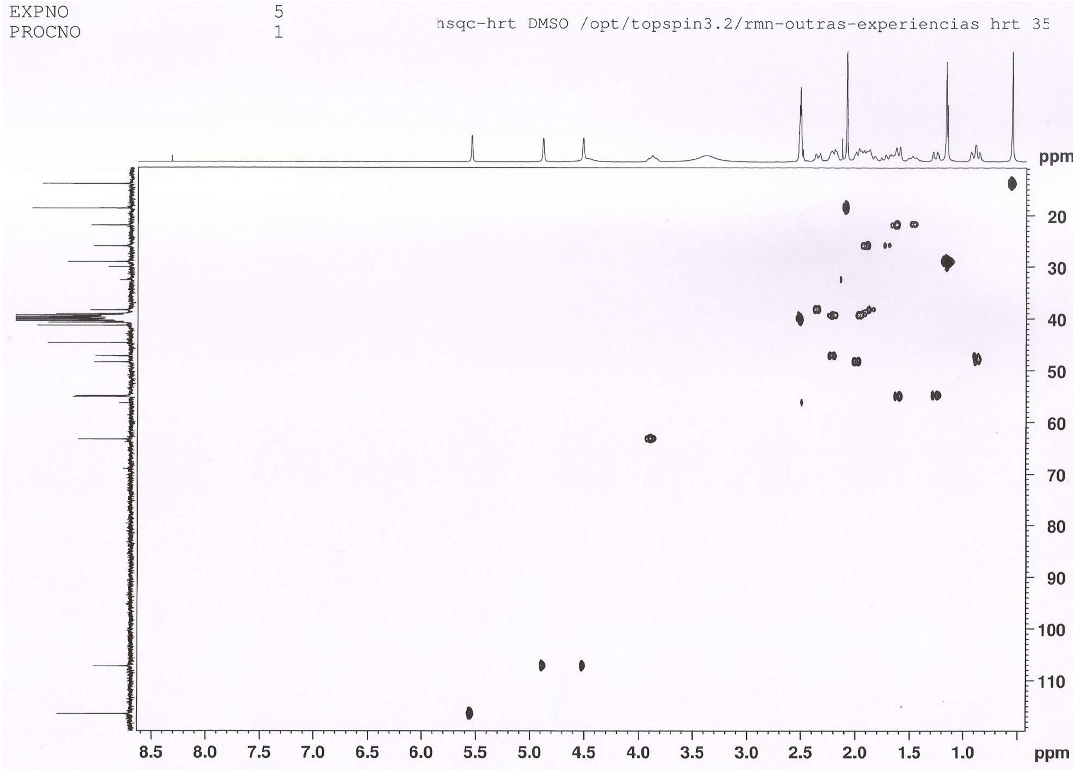

Figure S16. HMBC spectrum of **2b** (DMSOd6, 300 MHz).

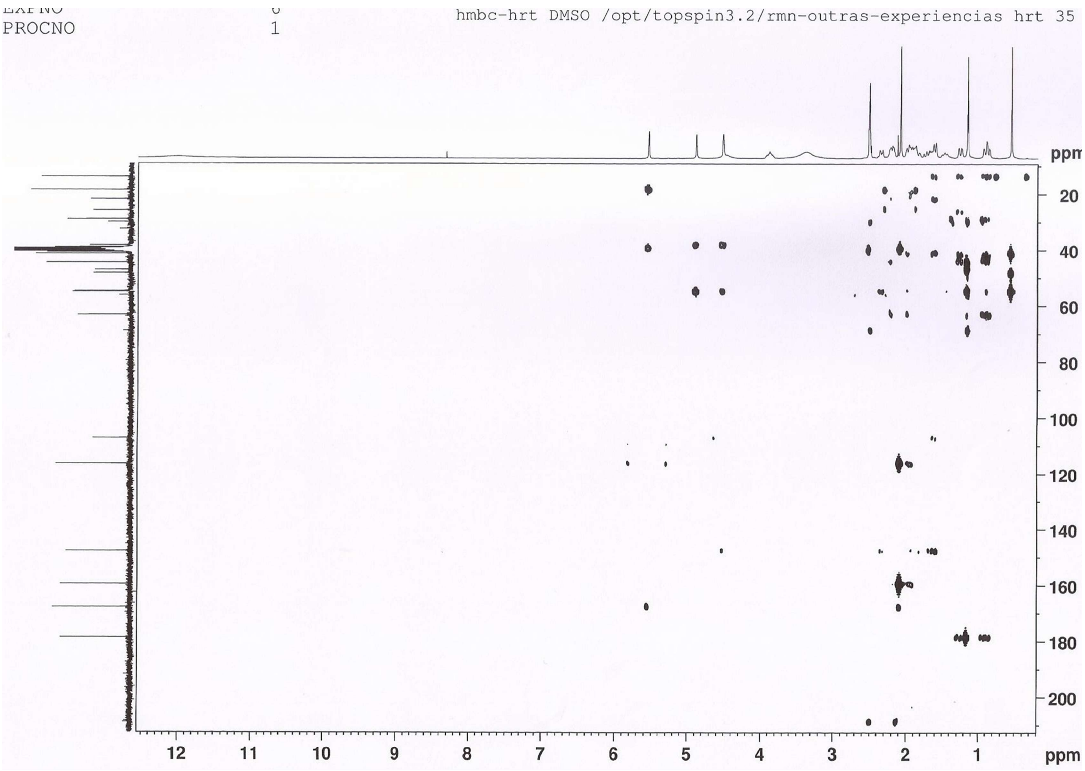

Figure S17. ROESY spectrum of **2b** ($DMSO_{d6}$, 300 MHz).

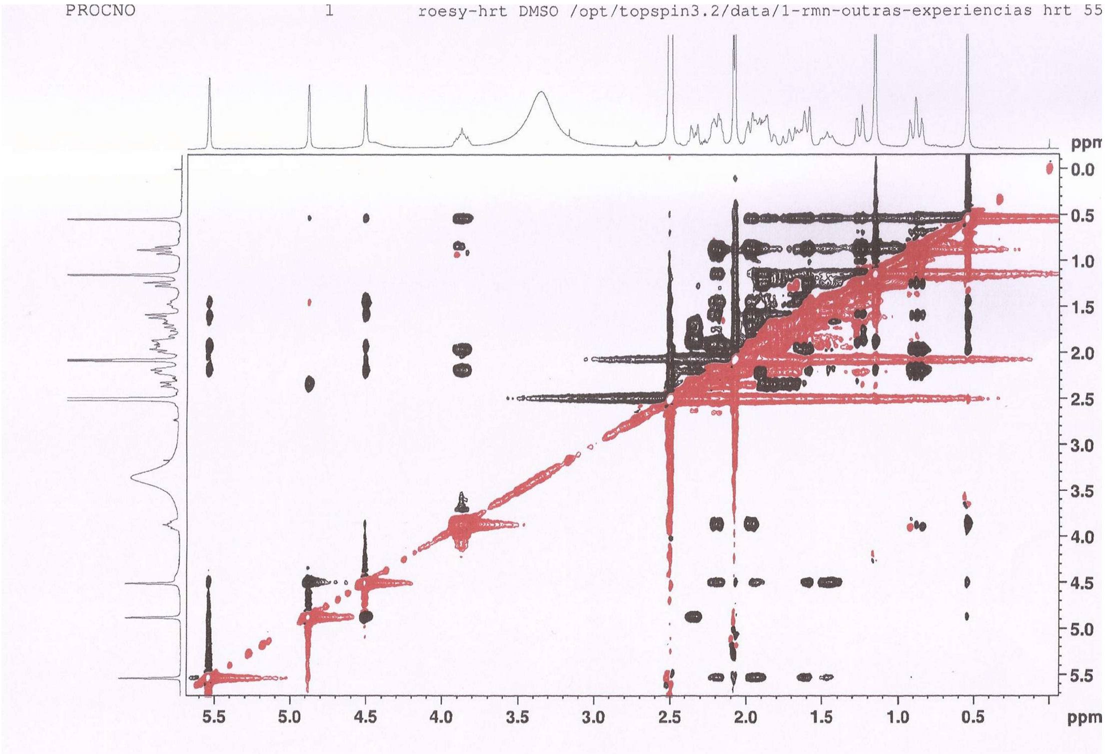

Figure S18. ^{1}HNMR spectrum of **3** ($DMSO_{d6}$, 300 MHz).

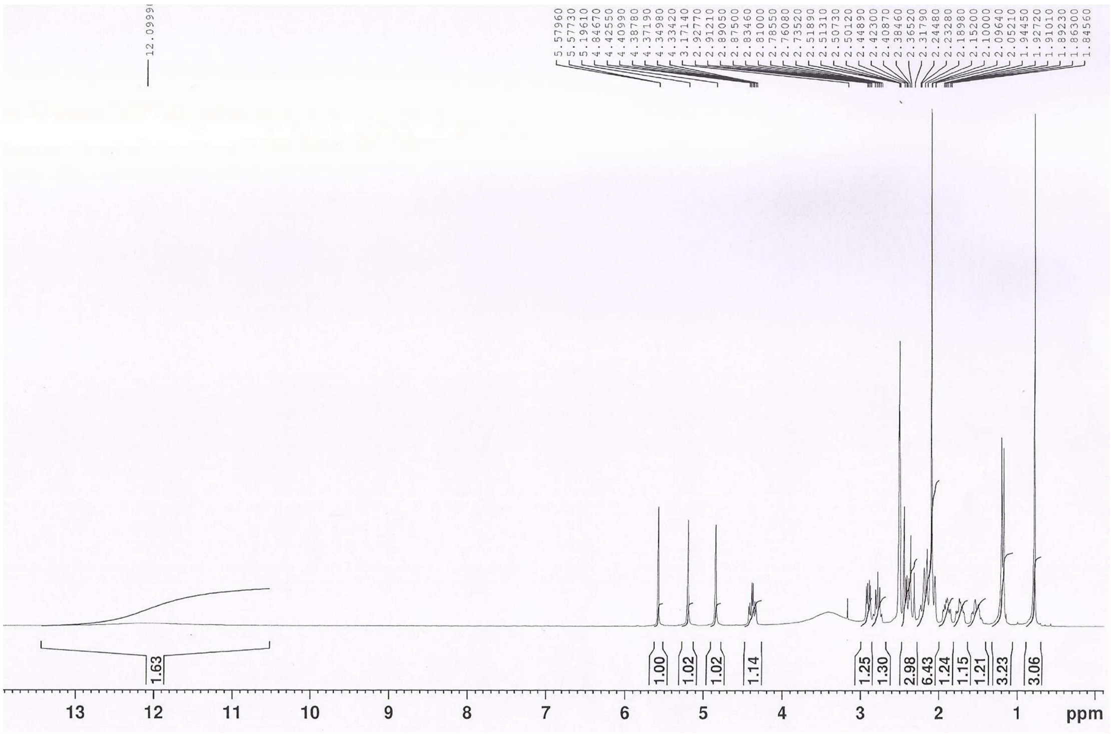

Figure S19. ^{13}C NMR spectrum of **3** ($DMSO_{d6}$, 75 MHz).

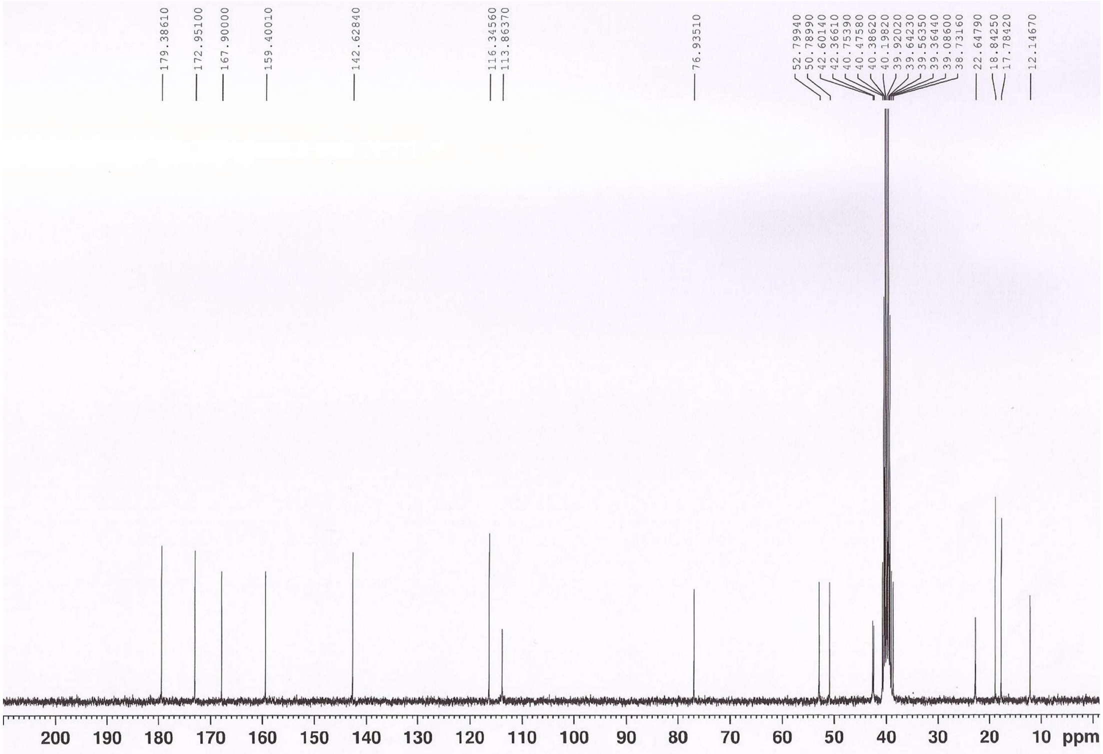

Figure S20. COSY spectrum of **3** ($DMSO_{d6}$, 300 MHz).

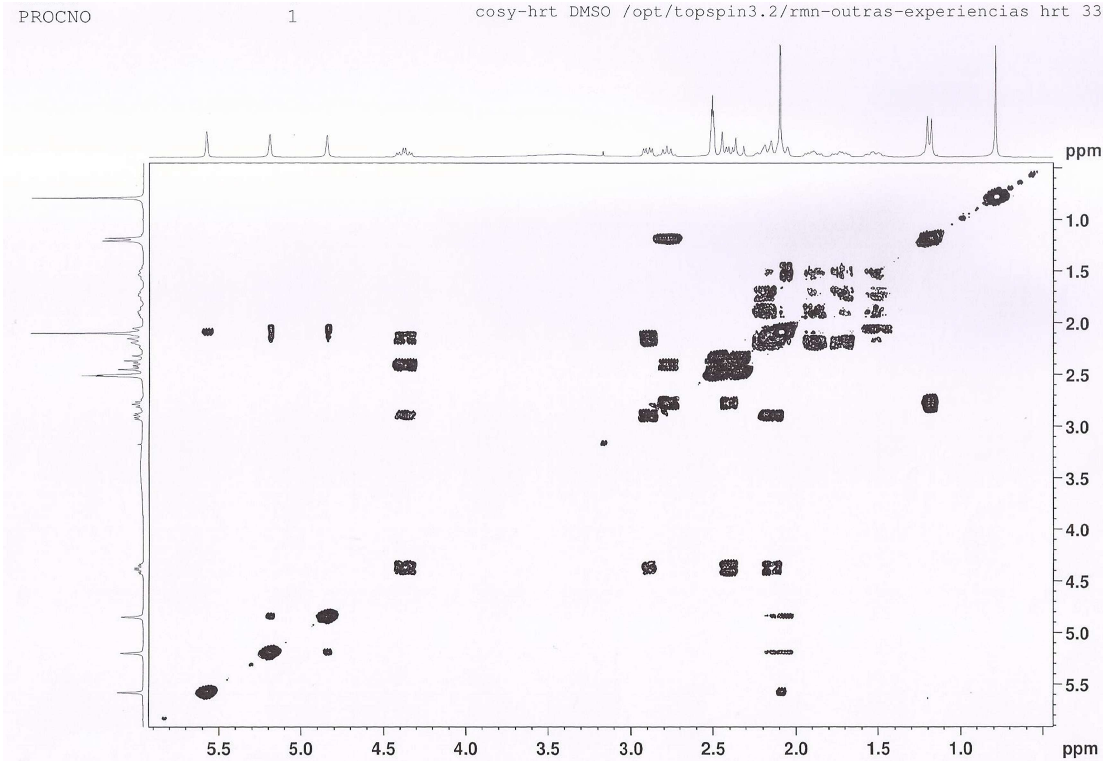

Figure S21. HSQC spectrum of **3** (DMSO$_{d6}$, 300 MHz).

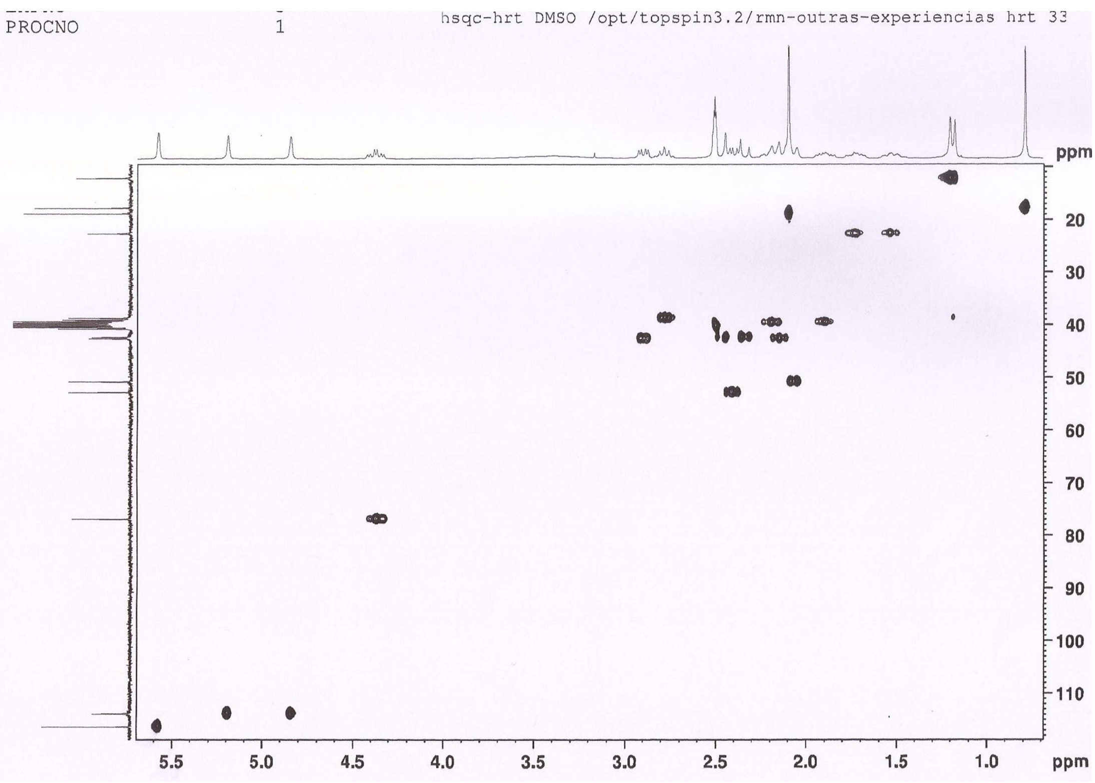

Figure S22. HMBC spectrum of **3** (DMSO$_{d6}$, 300 MHz).

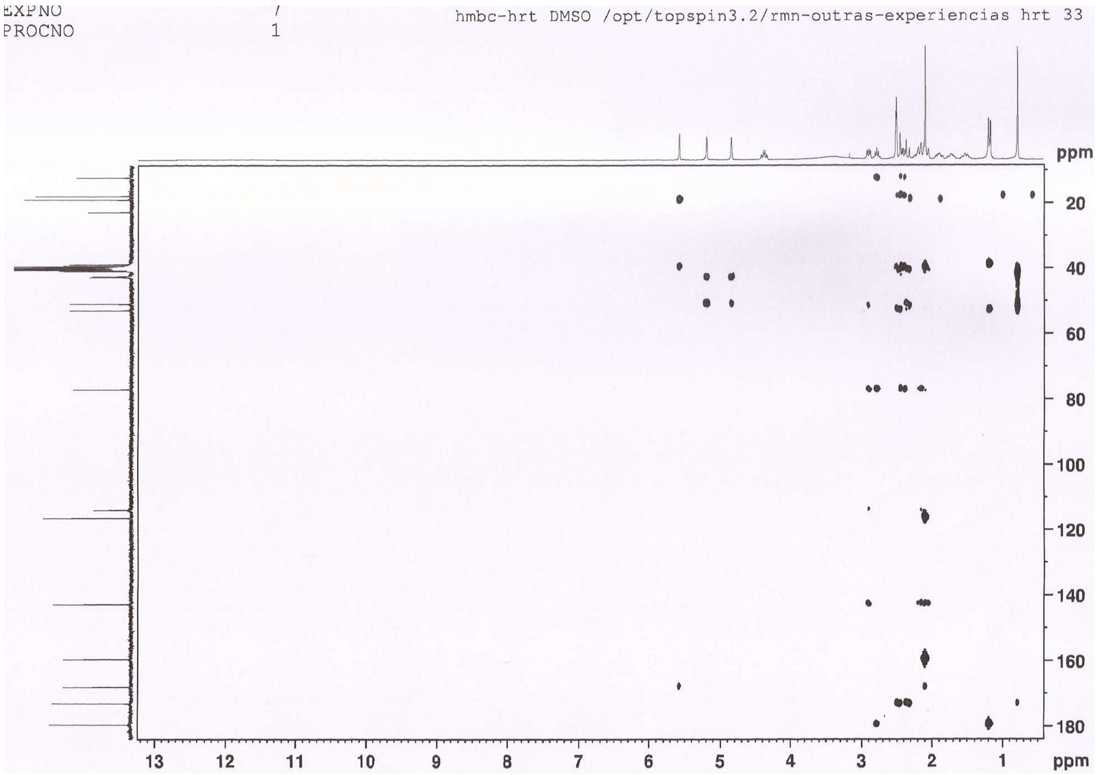

Figure S23. ^{1}H NMR spectrum of **4** ($DMSO_{d6}$, 300 MHz).

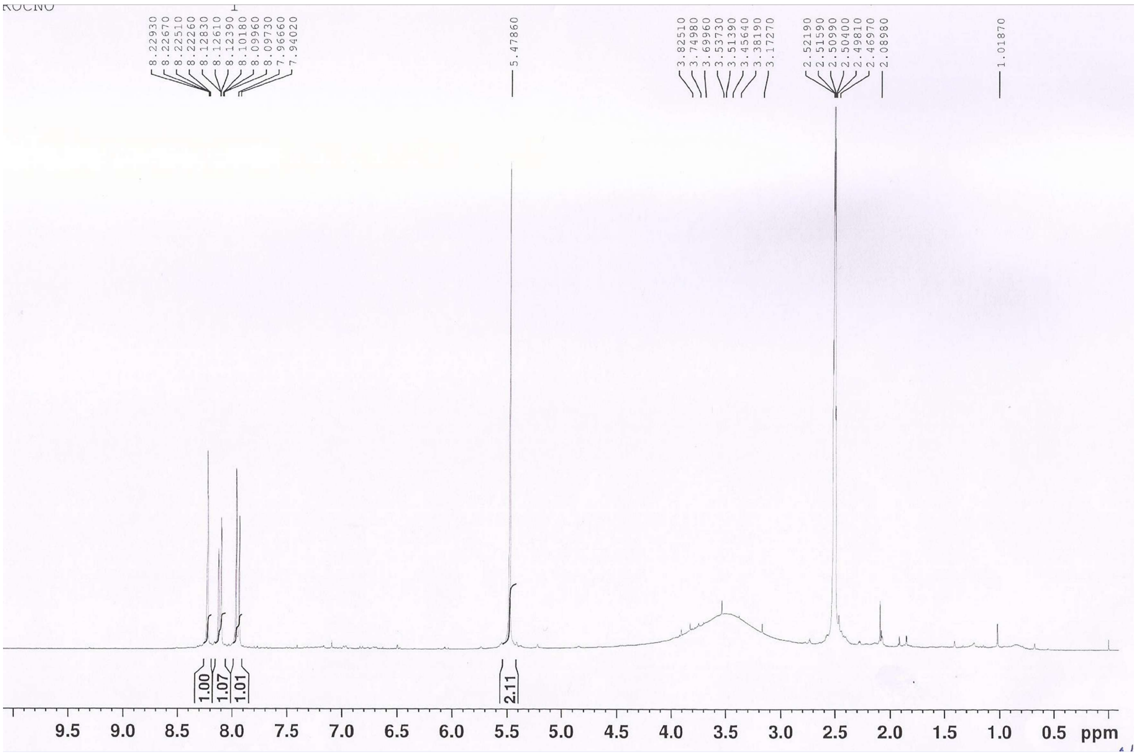

Figure S24. ^{13}C NMR spectrum of **4** ($DMSO_{d6}$, 75MHz).

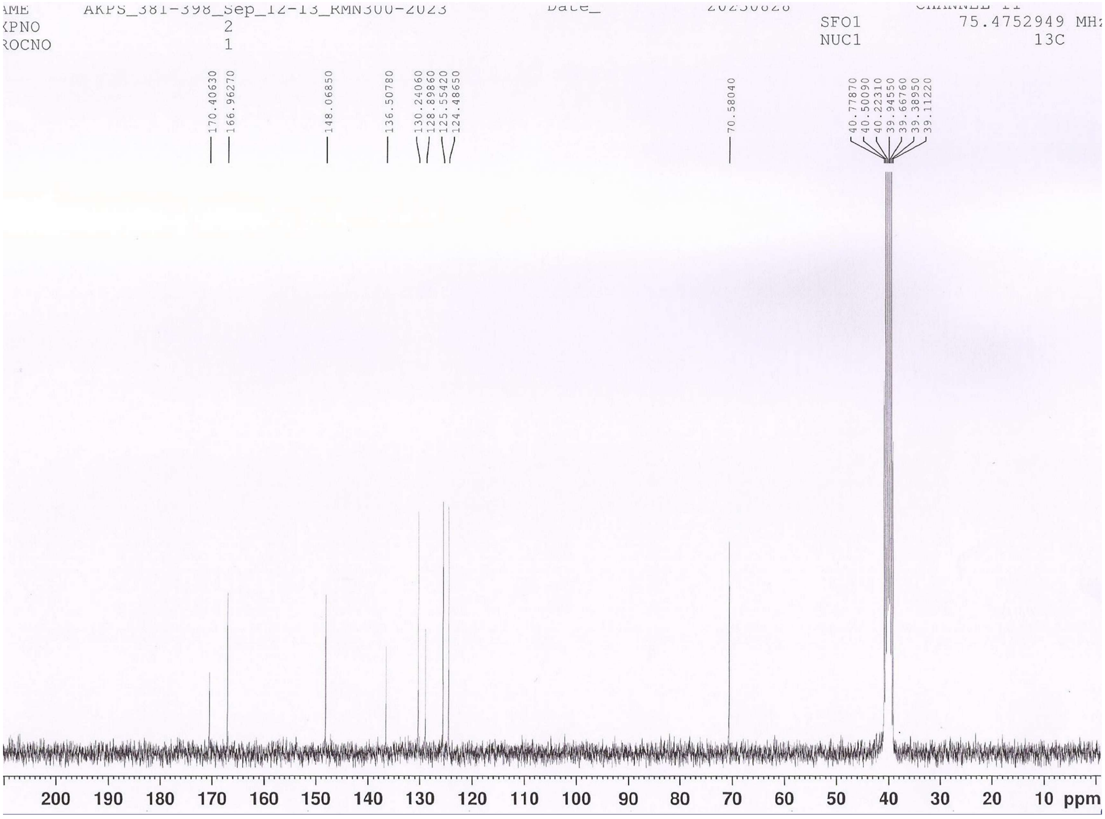

Figure S25. COSY spectrum of **4** (DMSO$_{d6}$, 300 MHz).

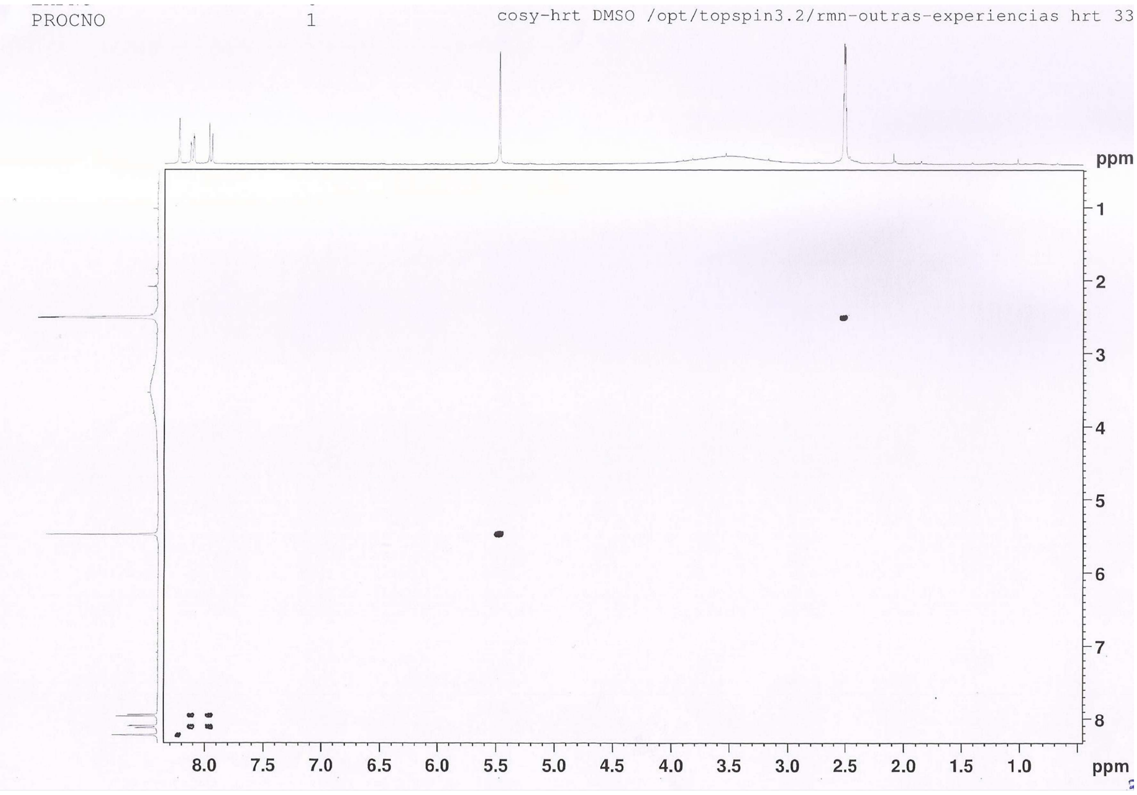

Figure S26. HSQC spectrum of **4** (DMSO$_{d6}$, 300 MHz).

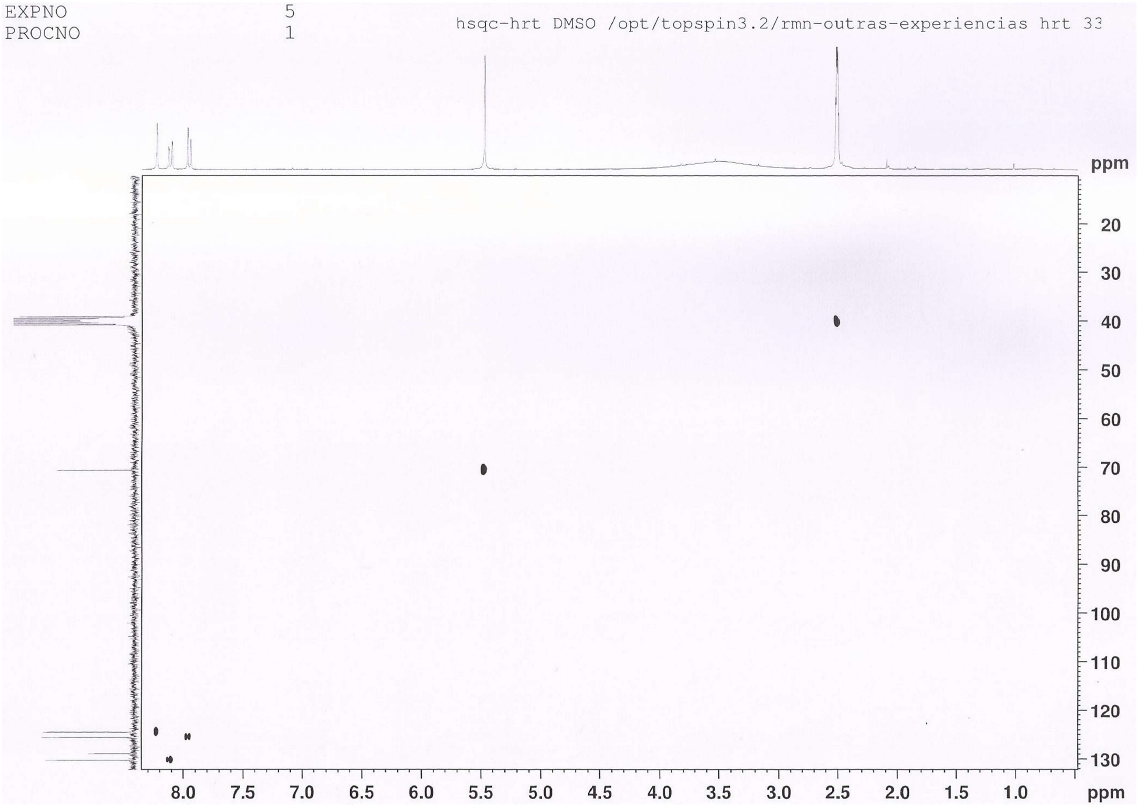

Figure S27. HMBC spectrum of **4** ($DMSO_{d6}$, 300 MHz).

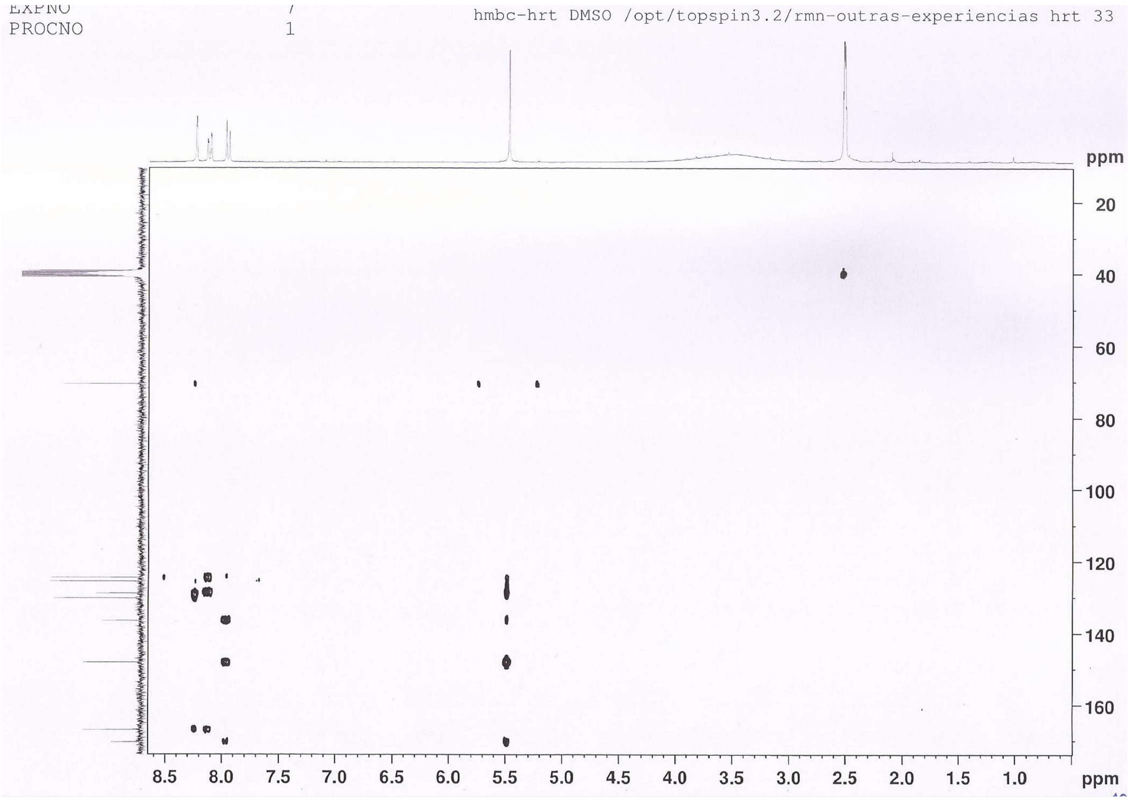

Figure S 28. (+)-HRESIMS of **1.**

Elemental Composition Report [MH]$^+$ (Match for $C_{15}H_{22}O_3$)
Single Mass Analysis
Tolerance = 5.0 PPM / DBE: min = -1.5, max = 100.0
Element prediction: Off
Number of isotope peaks used for i-FIT = 3
Monoisotopic Mass, Even Electron Ions
16 formula(e) evaluated with 1 results within limits (all results (up to 1000) for each mass)
Elements Used:
C: 15-15 H: 0-1050 O: 0-30

Minimum:				-1.5				
Maximum:		5.0	5.0	100.0				
Mass	Calc. Mass	mDa	PPM	DBE	i-FIT	Norm	Conf(%)	Formula
251.1654	251.1647	0.7	2.8	4.5	611.2	n/a	n/a	C15 H23 O3

Elemental Composition Report [MH]$^+$ (Match for $C_{15}H_{20}O_2$)
Single Mass Analysis
Tolerance = 5.0 PPM / DBE: min = -1.5, max = 100.0
Element prediction: Off
Number of isotope peaks used for i-FIT = 3
Monoisotopic Mass, Even Electron Ions
15 formula(e) evaluated with 1 results within limits (all results (up to 1000) for each mass)
Elements Used:
C: 15-15 H: 0-1050 O: 0-30

Minimum:				-1.5				
Maximum:		5.0	5.0	100.0				
Mass	Calc. Mass	mDa	PPM	DBE	i-FIT	Norm	Conf(%)	Formula
233.1550	233.1542	0.8	3.4	5.5	321.2	n/a	n/a	C15 H21 O2

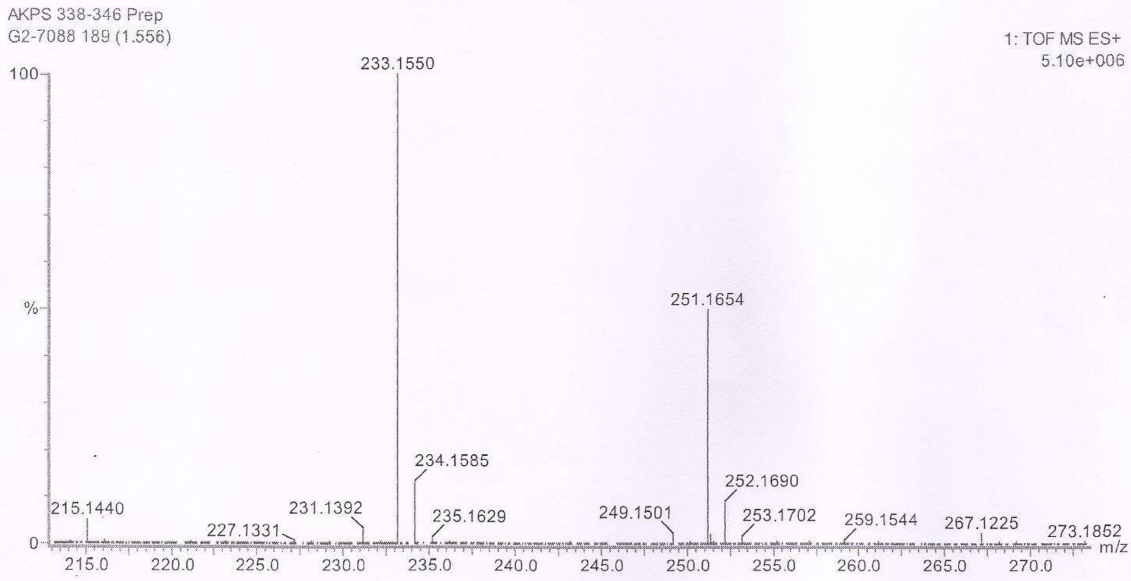

Supplement: Supplementary file 1 [file marinedrugs-24-00205-s001.zip › marinedrugs-4355203-supplementary.pdf]
